# Nuclear proteasomes buffer cytoplasmic proteins during autophagy compromise

**DOI:** 10.1038/s41556-024-01488-7

**Published:** 2024-08-29

**Authors:** So Jung Park, Sung Min Son, Antonio Daniel Barbosa, Lidia Wrobel, Eleanna Stamatakou, Ferdinando Squitieri, Gabriel Balmus, David C. Rubinsztein

**Affiliations:** 1 Department of Medical Genetics, and Cambridge Institute for Medical Research (CIMR), University of Cambridge, Cambridge, UK; 2 UK Dementia Research Institute, Cambridge Institute for Medical Research (CIMR), University of Cambridge, Cambridge, UK; 3 Huntington and Rare Diseases Unit, Fondazione IRCCS Casa Sollievo della Sofferenza Research Hospital, San Giovanni Rotondo, Italy; 4 UK Dementia Research Institute at University of Cambridge, Department of Clinical Neurosciences, University of Cambridge, Cambridge, UK; 5 Department of Molecular Neuroscience, Transylvanian Institute of Neuroscience, Cluj-Napoca, Romania

**Keywords:** Macroautophagy, Diseases

## Abstract

Autophagy is a conserved pathway where cytoplasmic contents are engulfed by autophagosomes, which then fuse with lysosomes enabling their degradation. Mutations in core autophagy genes cause neurological conditions, and autophagy defects are seen in neurodegenerative diseases such as Parkinson’s disease and Huntington’s disease. Thus, we have sought to understand the cellular pathway perturbations that autophagy-perturbed cells are vulnerable to by seeking negative genetic interactions such as synthetic lethality in autophagy-null human cells using available data from yeast screens. These revealed that loss of proteasome and nuclear pore complex components cause synergistic viability changes akin to synthetic fitness loss in autophagy-null cells. This can be attributed to the cytoplasm-to-nuclear transport of proteins during autophagy deficiency and subsequent degradation of these erstwhile cytoplasmic proteins by nuclear proteasomes. As both autophagy and cytoplasm-to-nuclear transport are defective in Huntington’s disease, such cells are more vulnerable to perturbations of proteostasis due to these synthetic interactions.

## Abstract (web summary)

Park et al. show that cells with impaired autophagy shuttle cytoplasmic proteins to the nucleus for degradation by nuclear proteasomes, revealing synergistic vulnerabilities in diseases where autophagy and nucleocytoplasmic transport are compromised.

## Main

Autophagy is a pathway conserved from yeast to humans enabling the degradation of cytoplasmic proteins and organelles. After such substrates are captured by autophagosomes, these are trafficked to lysosomes where their contents are degraded. Mutations in different core autophagy genes cause neurological syndromes, and autophagy compromise is a feature of neurodegenerative diseases such as Parkinson’s disease (PD) and Huntington’s disease (HD)^1,2^. Indeed, induction of autophagosome biogenesis lowers the levels of disease-causing proteins and ameliorates phenotypes in murine and other animal models of HD and related neurodegenerative diseases^3,4^. Given the importance of autophagy in health and disease, it is crucial to understand the processes that are buffered by normal autophagy and the cellular pathway perturbations that autophagy-perturbed cells are vulnerable to. We started to address this challenge by seeking to identify genetic interactions, when an allele of gene A combines with an allele of gene B resulting in a double-mutant with an unexpected phenotype^5–7^. This may be an exacerbation of the expected combined phenotype of the single-mutants; for example, two single-mutants have normal growth, but the double-mutant has slow growth, a negative interaction. The most severe negative interaction causing synthetic fitness loss is synthetic lethality, where two non-lethal single alleles combine to cause lethality, representing cellular pathways that buffer each other. This approach is suitable for identifying interactions with loss-of-function alleles of core autophagy genes, as these are well tolerated in mammalian cells^8,9^.

## Results

### Identifying genes that protect autophagy-defective cells

**Fig. 1:**
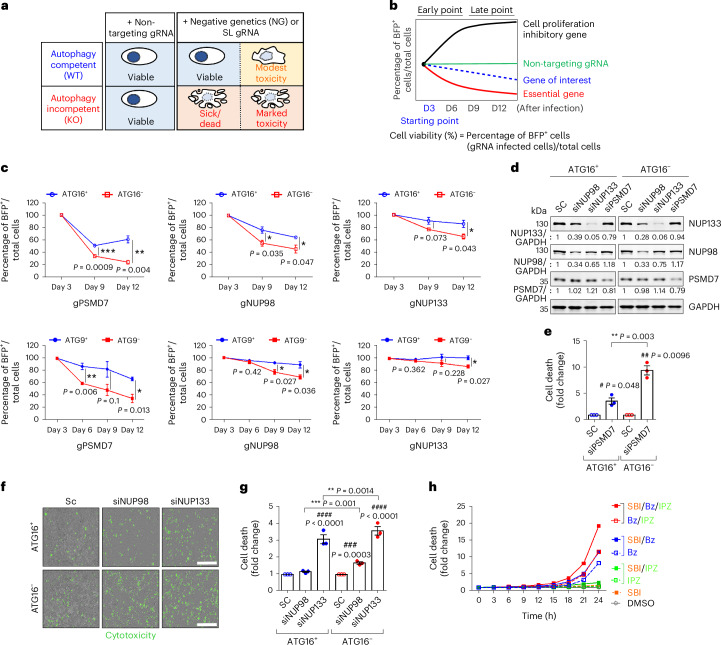
Identification of negative genetic interactors with autophagy compromise. **a**, Schematic representation of FACS-based CRISPR/Cas9 synthetic lethality (SL) screen to identify SL regulators required for survival during autophagy compromise. **b**, Quantification of cell viability assessed the percentage of BFP^+^ (blue fluorescent protein-positive (BFP), gRNA-infected) cells normalized to the number of total cells after transduction with gRNAs that are present in BFP^+^ cells, as the lentivirus vector carrying the gRNAs also expresses BFP. **c**, Quantification of cell viability with gRNAs for the indicated SL candidate genes (g*PSMD7*, g*NUP98* and g*NUP133*) in autophagy-incompetent cells (HeLa/*ATG16L1* knockout (KO) (ATG16^−^)/Cas9 (top), HeLa/*ATG9* KO (ATG9^−^)/Cas9 (bottom)) compared with their respective controls (autophagy-competent cells) (*n* = 3 technical triplicates after initial infected plates split into three plates of a 96-well plate for *ATG16L1* WT versus KO cells and *ATG9* WT versus KO cells. Percentage of BFP^+^ cells/total cells at 3 days set to 100% in all conditions to allow us to determine relative loss of cells over time in autophagy-competent versus autophagy-incompetent cells; two-tailed unpaired *t*-test). **d**, Immunoblots for SL candidate protein levels after each siRNA knockdown (representative blot from three biological repeats). **e**, Knockdown of PSMD7 enhances cell death in *ATG16L1* KO measured by LDH assay (day 3) (*n* = 3 independent experiments; two-tailed paired *t*-test). **f**, Illustration of readouts of Incucyte cell death assay (measured by Incucyte live-cell imaging). Scale bar, 600 μm. **g**, Quantification of CellTox Green fluorescence intensity after siRNA-mediated knockdown of NUP98 or NUP133 (day 5) versus scramble control siRNA (SC) (*n* = 3 independent experiments; two-way analysis of variance (ANOVA) with post hoc Tukey test). **h**, Combined effect of autophagy inhibition (SBI-0206965 (SBI) 5 μM) with proteasome inhibition (bortezomib (Bz) 1 μM) and/or nuclear import inhibition (importazole (IPZ) 2 μM) on HeLa cell death (Incucyte cell death assay with CellTox Green fluorescence). When we compute areas under the curve for four biological replicates, then SBI versus SBI + Bz, *P* = 0.013; SBI versus SBI + IPZ, *P* = 0.047 (one-tailed paired *t*-test). Values are mean ± s.e.m. For comparisons between ATG16^+^ and ATG16^−^ cell lines: * *P* < 0.05; ** *P* < 0.01; *** *P* = 0.001; for comparisons to relevant ATG16^+^ and ATG16^−^ control within cell line: # *P* < 0.05, ## *P* < 0.01, ### *P* < 0.001, #### *P* < 0.0001. Source numerical data and unprocessed blots are available in source data. Source data

**Extended Data Fig. 1:**
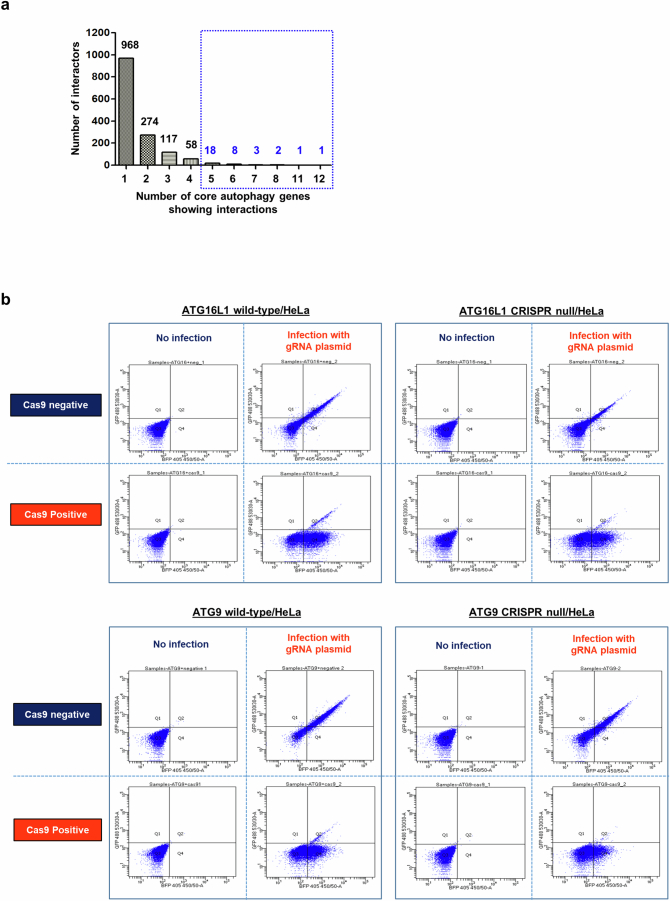
Synthetic lethality CRISPR/Cas9 screen. **a**, Numbers of human orthologues of yeast genes with negative genetic (NG) or synthetic lethal (SL) interactions with core autophagy genes, identified using databases (genetic interactions from YeastMine and human orthologues from HGCN) *in silico*. Number of core autophagy genes showing negative genetic/synthetic lethal interactions with specific genes is shown on x axis. **b**, Establishment of Cas9 stable lines in both HeLa/*ATG16L1* WT and *ATG16L1* KO (upper panel) and HeLa/*ATG9* WT and *ATG9* KO (lower panel) cells. Cells stably expressing Cas9 plasmid were selected. Cas9 enzyme cutting efficiency after gRNA plasmid infection was measured by FACS (flow cytometer) in *ATG16L1* WT and KO (upper panel) and in *ATG9* WT and KO (lower panel). Cas9 stable cell line (the pool) was assessed for Cas9 cutting efficiency with a lentiviral vector encoding BFP, GFP and a sgRNA against GFP. The percentage of BFP + /GFP- (edited) to BFP + /GFP+ (total transduced) cells was analysed in Cas9-negative and Cas9-positive cells by flow cytometer. (x axis shows BFP fluorescence, y-axis shows GFP fluorescence; Q1 (BFP-/ GFP+), Q2 (BFP + /GFP+), Q3 (BFP-/GFP-), and Q4 (BFP + /GFP-)). Source numerical data are available in source data. Source data

**Extended Data Fig. 2:**
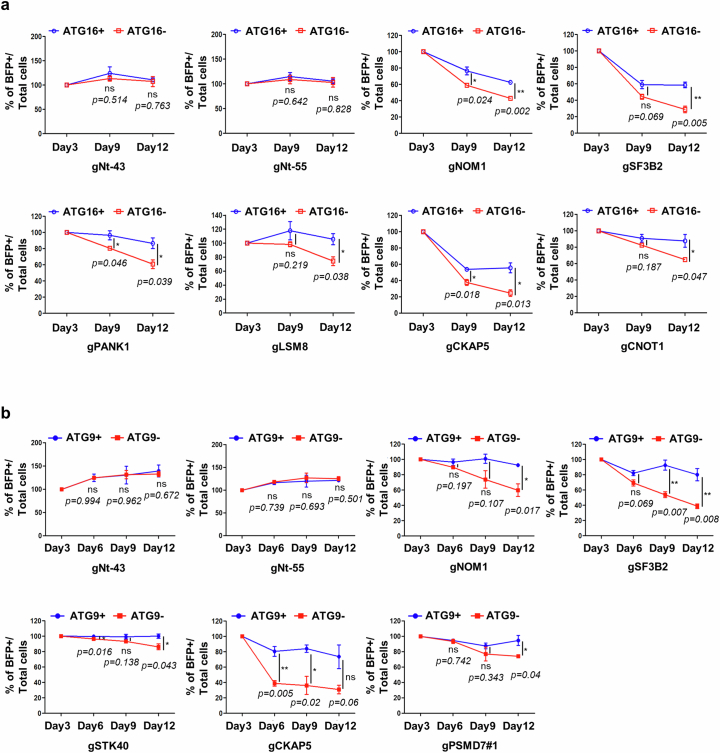
Results of synthetic lethality CRISPR/Cas9 screen in either autophagy-competent or -incompetent cells. **a, b**, Potential candidates from synthetic lethality (SL) CRISPR/Cas9 screen in *ATG16L1* wild-type (WT) (ATG16+) and knockout (KO) (ATG16-) (**a**) and in *ATG9* WT (ATG9+) and KO (ATG9-) cells (**b**) assessed at different time points after gRNA transduction. Two non-targeting gRNA sequences (gNt-43 and gNt-55) were employed as negative controls in the experiment. Values are mean ± S.E.M. (n = 3 technical triplicates after initial infected plates split into 3 plates of 96-well plate for *ATG16L1* WT vs. KO cells and *ATG9* WT vs. KO cells. % of BFP+ cells/total cells at 3 days (Day 3) set to 100 % in all conditions to allow us to determine relative loss of cells over time in autophagy-competent versus autophagy-incompetent cells; ns = not significant, * p < 0.05, ** p < 0.01; two-tailed unpaired t-test). Significantly decreased % of BFP+ cells indicating gRNA-infected cells (increased toxicity) in autophagy-null vs. WT cells indicates negative genetic/synthetic lethal interactions. Source numerical data are available in source data. LSM8 was only analysed in ATG16+ and ATG16- cells and was not pursued further in this study. Source data

**Extended Data Fig. 3:**
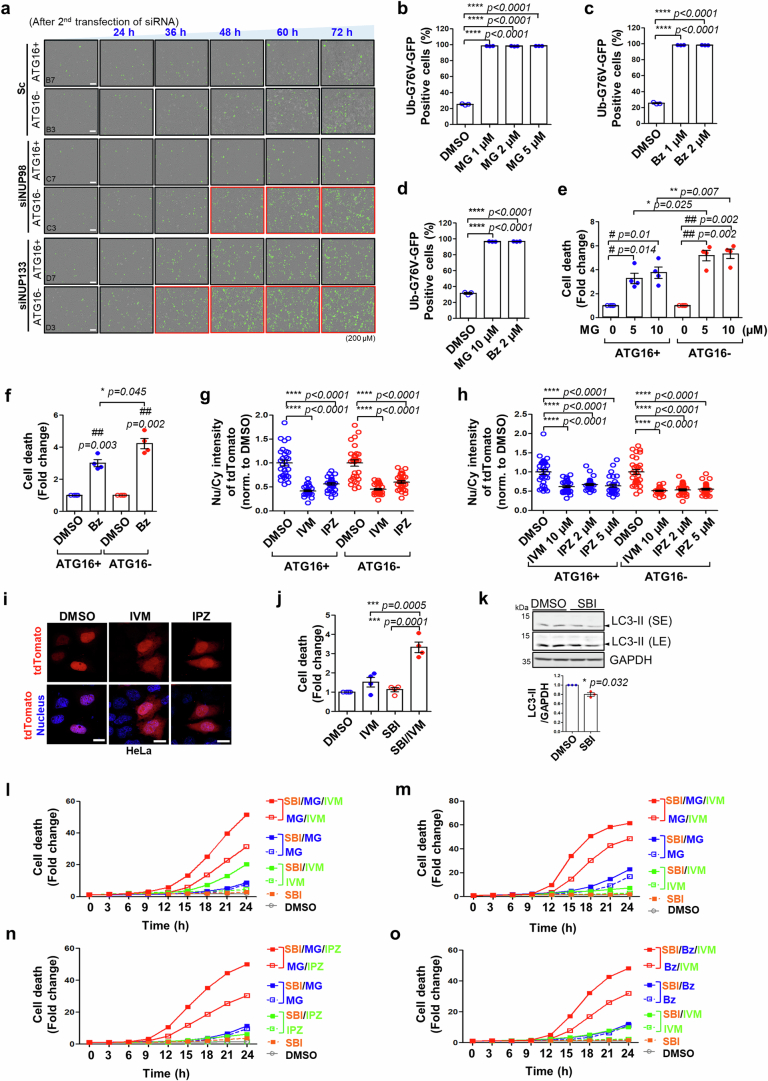
Autophagy inhibition enhances cell death upon proteasome inhibition and/or nuclear import inhibition. **a**, Representative images showing the cell death measured CellTox Green by real-time Incucyte in HeLa/*ATG16L1* wild-type (ATG16+) and null cells (ATG16-). Cells were double-transfected with siRNA against either Sc (Scrambled), NUP98 or NUP133, respectively, and stained with CellTox Green dye, then monitored by real-time Incucyte. Data in Day 5 (72 h after siRNA double transfection) is shown in Fig. 1f,g. Red boxes indicate times where there is synthetic lethality for the different siRNAs in ATG16-. Scale bar, 200 µm. **b-d**, Proteasome inhibitors (MG132 (MG) (**b, d**) and Bz (**c, d**)) inhibit proteasome activity measured by Ub-G76V-GFP, a ubiquitin fusion degradation (UFD) reporter, at different time points (**b-c**; 24 h and **d**; 6 h) and different concentrations in HeLa cells. (Value are mean ± S.E.M; n = 3 biological independent experiments; **** p < 0.0001 vs. DMSO; one-way ANOVA with post hoc Dunnett test) **e**, Effect of proteasome inhibitor MG132 (MG, for 24 h) on cell death measured by LDH assay in HeLa/*ATG16L1* WT (ATG16+) and KO (ATG16-) cells. Values are mean ± S.E.M. (n = 4 independent experiments; # p < 0.05, ## p < 0.01 vs. DMSO; * p < 0.05, ** p < 0.01 for relative changes induced by MG in WT vs. KO cells; two-tailed paired t-test). **f**, Effect of proteasome inhibitor Bortezomib (Bz, 1 µM for 24 h) on cell death in *ATG16L1* WT and KO. Cell death was detected by LDH assay. Values are mean ± S.E.M. (n = 4 independent experiments; ## p < 0.01 vs. DMSO; * p < 0.05 for relative changes induced by Bz in WT vs. KO cells; two-tailed paired t-test). **g-i**, HeLa cells were transfected with NLS–tdTomato-NES, a shuttling construct containing both an NLS and an NES fused to tdTomato (red). Cells were treated with nuclear import inhibitors (ivermectin (IVM) and importazole (IPZ)). (**g** and **h**) Quantification of ratio of nucleus/cytosol-localized tdTomato intensity in HeLa/*ATG16L1* wild-type and null cells at different concentrations and the different time points (IVM 25 µM and IPZ 25 µM for 6 h (**g**); IVM 10 µM, IPZ 2, 5 µM for 15 h (**h**) – data from one control experiment to confirm that the nuclear import inhibitors indeed affect this process) (Values are mean ± S.E.M; n = 30 cells in each condition with different time points and different concentrations; **** p < 0.0001 vs. DMSO; one-way ANOVA with post hoc Dunnett test). **i**, Representative pictures showing that nuclear import inhibitors (IVM 25 µM and IPZ 25 µM for 6 h) cause mislocalization of the NLS–tdTomato-NES to the cytoplasm in HeLa cells. Scale bar, 20 μm. These nuclear import inhibitors clearly increased the cytoplasmic pool of the NLS–tdTomato-NES reporter in steady-state – the failure to induce complete nuclear exclusion is consistent with other studies using these compounds with other NLS-containing substrates^14,55,56^ but may also reflect incomplete activities of the inhibitors at these concentrations^55,57^ and/or the possibility of a weakly functional NES in this reporter construct. **j**, The effect of autophagy inhibition (SBI, 5 µM for 24 h) combined with nuclear import inhibition by ivermectin (IVM, 10 µM for 24 h) on cell death measured by CellTox Green in HeLa cells. Values are mean ± S.E.M. (n = 4 independent experiments; *** p < 0.001 for relative changes induced by SBI (autophagy inhibition) or IVM (nuclear import inhibition) vs. combined inhibition; one-way ANOVA with post hoc Dunnett test). **k**, Effect of autophagy/ULK1 inhibitor SBI-0206965 (SBI) on LC3-II levels which correlated with autophagosome numbers in HeLa cells. Quantified data represents autophagy inhibition by SBI on LC3-II level. Blots are representative of three biologically independent experiments. Values are mean ± S.E.M. (* p < 0.05 vs. DMSO; two-tailed paired t-test). **l-o**, Combined effect of autophagy inhibition with proteasome and/or nuclear import inhibition on cell death were measured by real-time cell death assay (CellTox Green) at 3 h intervals. HeLa cells were treated with indicated distinct inhibitors in growth media with CellTox Green dye for 24 h. Cell death were measured with CellTox Green fluorescence/total area by Incucyte live-cell imaging. (**l**) Autophagy inhibition (SBI, 5 µM), proteasome inhibition (MG132 (MG), 2 µM), nuclear import inhibition (Ivermectin (IVM), 10 µM). (**m**) Autophagy inhibition (SBI, 5 µM), proteasome inhibition (MG, 5 µM), nuclear import inhibition (IVM, 10 µM). (**n**) Autophagy inhibition (SBI, 5 µM), proteasome inhibition (MG, 5 µM), nuclear import inhibition (Importazole (IPZ), 5 µM). (**o**) Autophagy inhibition (SBI, 5 µM), proteasome inhibition (Bortezomib (Bz), 1 µM), nuclear import inhibition (IVM, 10 µM). Autophagy inhibition plus proteasome inhibitor and/or nuclear import inhibitor stimulates more cell death than each inhibition alone. Each graph of cell death represents same order of toxicity. Source numerical data and unprocessed blots are available in source data. Source data

As synthetic lethal interactions between yeast genes increase the likelihood of such interactions between their human orthologues 3–19-fold^10–12^, we analysed human orthologues of yeast genes with at least five negative genetic or synthetic lethal interactions with core autophagy genes, identified using two databases (from genetic interactions available in YeastMine and from their orthologues available in HGCN) (Supplementary Table 1 and Extended Data Fig. 1a). We used this cutoff to maximize the chance that the interactions were via autophagy and not through autophagy-independent functions of autophagy genes. We used CRISPR/Cas9 to knock out each of the 30 human orthologues and compared the survival of these knockouts in *ATG16L1* or *ATG9* wild-type (WT) (*ATG16*^*+*^ or *ATG9*^+^) versus isogenic *ATG16L1* knockout (*ATG16*^*−*^) or *ATG9* knockout (*ATG9*^−^) HeLa cells, which lack key genes required for autophagosome biogenesis (Fig. 1a and Extended Data Fig. 1b)^9,13^. Note that the *ATG16L1*-null and *ATG9*-null cells, which have normal viability, each have their own control lines. Our fluorescence-activated cell sorting (FACS)-based cell number assay with CRISPR/Cas9 knockouts of human orthologues of yeast genes showing synthetic lethality with autophagy gene knockouts identified potential synthetic lethal interactions with both *ATG16L1*-null and *ATG9*-null cells. These were selected on the basis of at least one out of two guides tested showing a more rapid decline in cell numbers in both autophagy-null lines versus their matched WT, autophagy-competent control lines: *PSMD7* (proteasome 26S subunit, non-ATPase 7), *NUP98* (nucleoporin 98 and 96 precursor) and *NUP133* (nucleoporin 133) (Fig. 1b,c, Extended Data Fig. 2 and Supplementary Tables 2 and 3). We validated these candidates implicating the proteasome and nuclear pore complex (NPC) as synthetic lethal interactors with autophagy by assessing cell death in *ATG16L1*-null (ATG16^−^) versus WT (ATG16^+^) cells using siRNAs as distinct reagents (Fig. 1d–g and Extended Data Fig. 3a). To follow-up *PSMD7*, we further confirmed that different proteasome inhibitors (MG132 and bortezomib) enhanced cell death in *ATG16L1*-null versus WT cells (Extended Data Fig. 3b–f). Similarly, we observed a dramatic increase in cell death when we combined ivermectin^14^, an importin α/β inhibitor, with SBI-0206965 (SBI), a highly selective autophagy kinase *ULK1* inhibitor, versus either compound alone (Extended Data Fig. 3g–k)^15^. In time-course studies, we also found that autophagy inhibition alone (SBI) was less toxic than autophagy inhibition plus proteasome inhibition by bortezomib and/or nuclear import inhibition by importazole, which is an importin β inhibitor^16^ (Fig. 1h). Similar trends were seen in time-course studies with distinct proteasome and nuclear import inhibitors (MG132 and ivermectin^14^, respectively) at different concentrations and combinations (Extended Data Fig. 3l–o). Moreover, consistent with these candidates, other proteasome subunits and nucleoporins were identified in yeast as negative genetic and synthetic lethal interactors with four or fewer distinct autophagy core gene knockouts (Supplementary Table 4). Taken together, these data suggest that nucleoporins and proteasome activity buffer cell survival in autophagy-null cells and that these genetic interactions are conserved from yeast to mammalian cells.

### Nuclear relocation of substrates in autophagy compromise

**Fig. 2:**
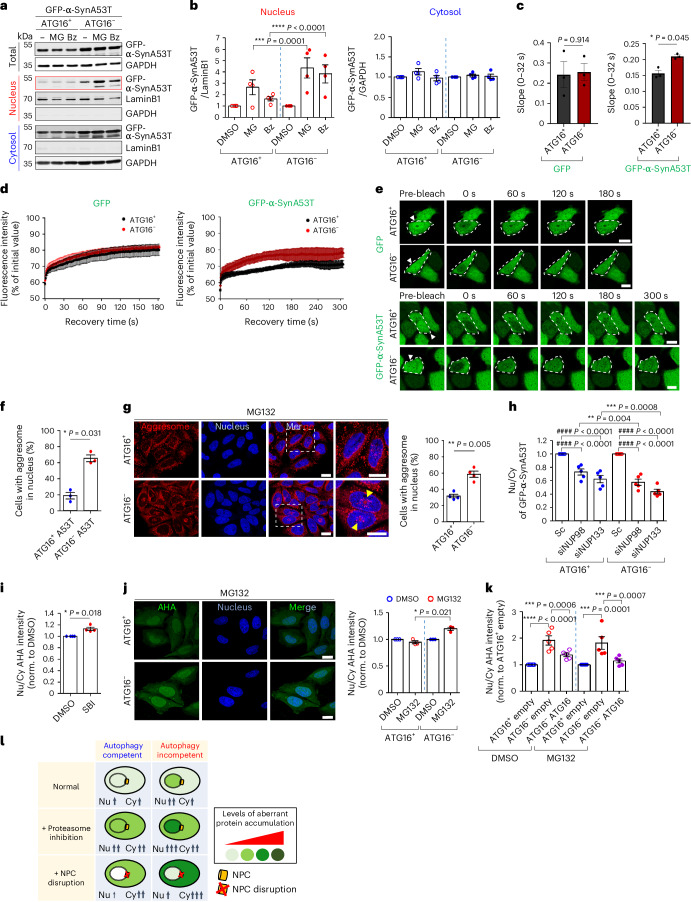
Autophagy depletion causes nuclear translocation of erstwhile cytoplasmic autophagic substrates. **a**, Autophagy substrate A53T α-Syn localized more in the nucleus of *ATG16L1* KO (ATG16^−^) cells compared with *ATG16L1* WT (ATG16^+^) cells treated with proteasome inhibitor (MG132 (MG, 10 μM, 6 h) or Bz (2 μM, 6 h)) by cell fractionation. **b**, Quantification of western blots (representative in **a**) showing relative changes of A53T α-Syn in nucleus and cytosol within cell lines caused by proteasome inhibitors (DMSO = 1) (*n* = 4 independent experiments; two-way ANOVA with post hoc Tukey test). **c**–**e**, Nuclear FRAP in *ATG16L1* WT and KO cells expressing either GFP-empty or GFP-A53T α-Syn. The initial recovery slope (0–32 s) rate of **d** (*n* = 3 independent experiments; two-tailed paired *t*-test) (**c**). Nuclear FRAP curves show faster recovery of GFP-A53T α-Syn in *ATG16L1* KO cells compared with WT cells, while this effect was not seen with GFP-empty (**d**). Representative images of *ATG16L1* WT and KO cells expressing either GFP-empty or GFP-A53T α-Syn before and after photobleaching and after recovery (up to 3 min or 5 min) (**e**). Arrowhead indicates photobleached cell. Scale bar, 10 µm. **f**, Increased nuclear aggresomes by Proteostat dye in *ATG16L1* WT and KO cells expressing A53T α-Syn treated with MG (*n* = 3 independent experiments; two-tailed paired *t*-test). **g**, Increased nuclear aggresomes in *ATG16L1* KO compared with WT with MG (2 μM, 15 h) (*n* = 4 independent experiments; two-tailed paired *t*-test). Scale bar, 20 µm. **h**, Knockdown of NUP98 or NUP133 inhibits A53T α-Syn shuttling into nucleus in *ATG16L1* KO cells compared with WT cells, by immunostaining (*n* = 5 independent experiments; two-way ANOVA with post hoc Tukey test). **i**,**j**, Localization of AHA-labelled proteins by immunostaining upon either autophagy inhibition with SBI (5 µM, 15 h) in HeLa (**i**) (*n* = 4) or proteasome inhibition (MG, 2 µM, 15 h) in *ATG16L1* WT and KO cells (**j**) (*n* = 3 independent experiments; two-tailed paired *t*-test). Scale bar, 20 µm. **k**, Overexpression of ATG16L1 rescues mislocalization of AHA-labelled proteins in *ATG16L1* KO cells by western blotting (*n* = 5 independent experiments; one-way ANOVA with post hoc Tukey test). **l**, Schematic representation shows idealized protein levels/localization upon proteasome inhibition or NPC disruption in WT and autophagy-null cells. Values are mean ± s.e.m. Source numerical data and unprocessed blots are available in source data. DMSO, dimethylsulfoxide. Source data

**Extended Data Fig. 4:**
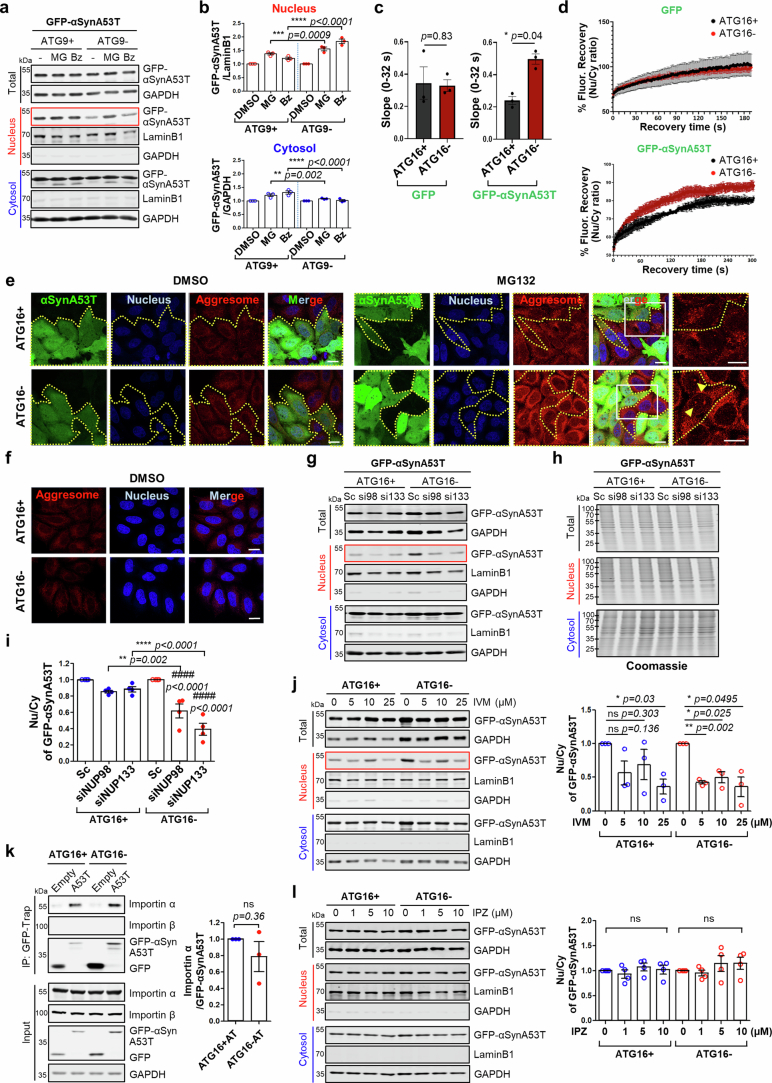
Autophagy inhibition causes autophagic substrate shuttling into the nucleus. **a, b**, Autophagy substrate αSyn A53T localizes in the nucleus more in *ATG9* KO (ATG9-) compared to WT (ATG9+) cells. Immunoblots for GFP-αSyn A53T localization in nucleus and cytosol fraction upon proteasome inhibition with MG132 (MG,10 µM) or bortezomib (Bz, 2 µM) for 6 h in *ATG9* WT and KO (**a**). (**b**) Quantification of relative changes of A53T α-Syn in nucleus and cytosol within *ATG9* WT and KO cells (DMSO = 1) induced by proteasome inhibitors. Values are mean ± S.E.M. (n = 3 independent experiments; ** p < 0.01, *** p < 0.001, **** p < 0.0001 for relative changes induced by proteasome inhibitors in WT vs. KO cells; two-way ANOVA with post hoc Tukey test). **c, d**, Nuclear fluorescence recovery after photobleaching (FRAP) in *ATG16L1* WT and KO cells expressing either GFP-empty or GFP-A53T α-Syn. FRAP was measured by bleaching the entire nucleus and monitoring fluorescence recovery up to 3 min for GFP or 5 min for GFP-A53T α-Syn, respectively, in line with the nuclear fluorescence recovery kinetics of these different proteins shown in Fig. 2c–e. **c** and **d**, Nuclear FRAP was determined as the change in ratio of Nucleus/Cytosol fluorescence, respectively, in cells where we examined a region of interest of the nucleus that had been previously photobleached and a region of the cytoplasm that had not been photobleached. **c**, The rate showing the initial recovery slope (0–32 s) of **d**. Values are mean ± S.E.M. (n = 3 independent experiments; * p < 0.01; two-tailed paired t-test). **d**, Mean FRAP curves showing that the change in ratio of Nucleus/Cytosol fluorescence of GFP-A53T αSyn in *ATG16L1* KO cells recovered faster than in WT cells, while this effect was not seen with GFP-empty. Values are mean ± S.E.M. (n = 3 independent experiments) **e**, Representative images show the effect of GFP-αSyn A53T on the number of nuclear-localized aggresomes upon proteasome inhibition described in Fig. 2f. Arrowhead indicates nuclear-localized aggresomes. Images are representative of three biologically independent experiments. Scale bar, 20 µm. **f**, Control data for Fig. 2g showing baseline aggresome staining in DMSO. Figure 2g shows MG132 effect. Images are representative of four biologically independent experiments in the condition of DMSO. Scale bar, 20 µm. **g-i**, Inhibition of nuclear pore complex (NPC) blocks autophagic substrate shuttling into nucleus more in *ATG16L1* KO cells compared to WT cells. **g**, Representative immunoblots for **i** showing GFP-αSyn A53T mutant localization in nucleus or cytosol fraction upon either NUP98 (si98) or NUP133 (si133) knockdown in ATG16L1 WT and KO. Blots are representative of four biologically independent experiments. **h**, Knockdown of NUP98 and NUP133 on total protein levels in nucleus or cytosol fraction by Coomassie staining. **i**, Values are mean ± S.E.M. (n = 4 independent experiments; #### p < 0.0001 vs. Sc; ** p < 0.01, **** p < 0.0001 for relative changes induced by specific siRNA in *ATG16L1* WT (blue dots) vs KO (red dots) cells; two-way ANOVA with post hoc Tuckey test). **j**, Representative immunoblots showing that for nuclear import inhibition by importin α/β inhibitor ivermectin (IVM) blocked αSyn A53T shuttling into the nucleus in ATG16L1 KO cells assessed by cell fractionation. (**right panel**) Quantified data represents the ratio of nuclear/cytosol-localized αSyn A53T. Values are mean ± S.E.M. (n = 3 independent experiments; ns = not significant, * p < 0.05, ** p < 0.01 vs. DMSO; two-tailed paired t-test). **k**, Representative blots showing the binding of αSyn A53T with importin α in *ATG16L1* KO cells compared to WT. Cells were transfected with either GFP-empty or GFP-αSyn A53T and then immunoprecipitates obtained using GFP-trap were processed for immunoblotting to detect importin α and importin β. (**right panel**) Quantification shows the amount of importin α -bound GFP-αSyn A53T (Values are mean ± S.E.M; n = 3 biological independent experiments; ns = not significant ATG16+ vs. ATG16- cells overexpressing GFP-αSyn A53T (AT); two-tailed paired t-test). **l**, Representative immunoblots showing that nuclear import inhibition by importin β inhibitor importazole (IPZ) did not affect αSyn A53T shuttling into the nucleus in *ATG16L1* KO cells assessed by cell fractionation. (**right panel**) Quantified data represents the ratio of nuclear/cytosol-localized αSyn A53T. Values are mean ± S.E.M. (n = 4 independent experiments; ns = not significant vs. DMSO; two-tailed paired t-test). Source numerical data and unprocessed blots are available in source data. Source data

The A53T α-synuclein (A53T α-Syn) mutant, which causes autosomal dominant PD, is cleared by both autophagy and the proteasome^17^, the two major intracellular clearance pathways. Thus, we have used this protein as a model substrate. We observed that short-term (6 h) proteasome inhibition by MG132 or bortezomib caused an accumulation of A53T α-Syn in the nucleus of autophagy-null versus WT cells (Fig. 2a,b and Extended Data Fig. 4a,b). Fluorescence recovery after photobleaching (FRAP) experiments confirmed that the green fluorescent protein (GFP)-A53T α-Syn (a good autophagy substrate) signal in the nucleus recovered more rapidly in *ATG16L1*-null versus WT cells and no such effect was seen with GFP alone (a poor autophagy substrate predominantly cleared by proteasome) (Fig. 2c–e shows just the nuclear FRAP recovery and Extended Data Fig. 4c,d shows the nuclear FRAP recovery divided by the fluorescence of an unbleached cytoplasmic region in the same cell exposed to nuclear photobleaching). These data further support the steady-state data suggesting that autophagy compromise leads to nuclear transport of A53T α-Syn (Fig. 2c–e and Extended Data Fig. 4c,d). MG132 treatment of A53T α-Syn-expressing cells also increased nucleus-localized aggregated proteins (aggresomes) detected with Proteostat dye in *ATG16L1*-null versus WT cells (Fig. 2f and Extended Data Fig. 4e). Furthermore, prolonged proteasome inhibition caused a greater proportion of autophagy-null cells (without A53T α-Syn) to exhibit nuclear accumulation of endogenous aggresomes, compared with proteasome-inhibited WT cells (Fig. 2g and Extended Data Fig. 4f). Thus, we hypothesized that autophagy inhibition, which impacts cytoplasmic protein degradation, causes relocation of substrates to the nucleus for degradation by nuclear proteasomes. Indeed, knockdown of either of the NPC constituents NUP98 (refs. ^18,19^) or NUP133 (ref. ^20^), two other hits from our synthetic lethal screen, as well as treatment with ivermectin, an importin α/β inhibitor^16^, decreased A53T α-Syn nuclear/cytoplasmic ratios specifically in autophagy-null cells by immunoblotting of fractionated cells (Extended Data Fig. 4g–j) and by microscopy (Fig. 2h). Notably, these data are consistent with published work showing that nuclear import of α-Syn is importin α-dependent, and associated with the interaction of importin α with α-Syn^21^, which we confirmed (Extended Data Fig. 4k). Importin α can directly bind to certain proteins and enable their nuclear import without the requirement of importin β, highlighting the versatility and complexity of nucleocytoplasmic transport mechanisms^22–24^. This non-canonical form of importin α-dependent, importin β-independent nuclear import is likely driven by the ability of importin α itself to enter the nucleus, independently of importin β^22^. However, α-Syn did not bind importin β, and this may explain why the importin β inhibitor importazole did not affect A53T α-Syn shuttling (Extended Data Fig. 4l). These data suggest that proteasome inhibition and impairment of NPC function may act via a common pathway to cause synergistic viability changes akin to synthetic fitness loss in autophagy-null cells.

### Autophagy inhibition relocates ‘bulk’ proteins into nucleus

**Extended Data Fig. 5:**
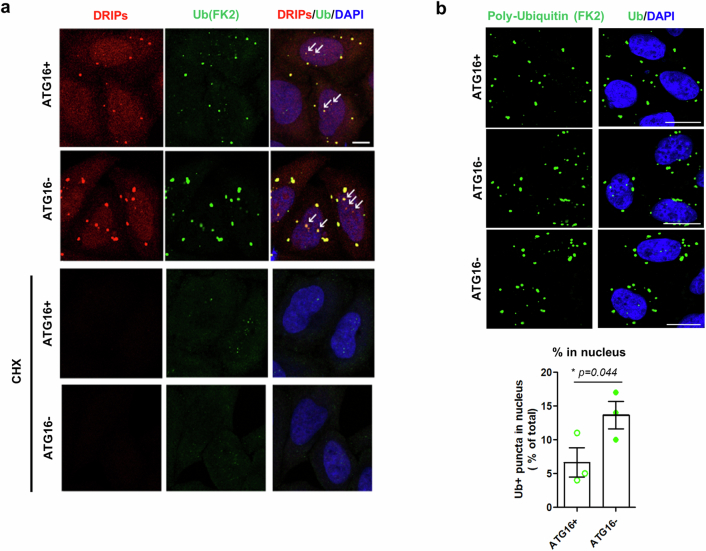
Puromycin-induced misfolded protein inclusions are found in the nucleus in ATG16L1 KO cells. **a**, *ATG16L1* WT (ATG16+) and KO (ATG16-) HeLa cells were treated with O-propargyl-puromycin (OP-Puro, DRIPs) with or without cycloheximide (CHX, 50 µg/ml) as a control for 2 h. Cells were fixed and OP-Puro and ubiquitin-positive structures were visualized using confocal microscopy. Arrow indicates DRIPs in the nucleus. Scale bar, 10 µm. (Representative images from 3 biological repeats) **b**, *ATG16L1* WT and KO HeLa cells were treated with puromycin for 4 h. Cells were fixed and ubiquitin-positive structures were visualized using confocal microscopy. Total area of ubiquitin-positive structures (Ub+) in nucleus and entire cell was quantified. Scale bar, 20 µm. Values are mean ± S.E.M. (n = 3 independent experiments; * p < 0.05 vs. WT cells; two-tailed paired t-test). Source numerical data are available in source data. Source data

**Extended Data Fig. 6:**
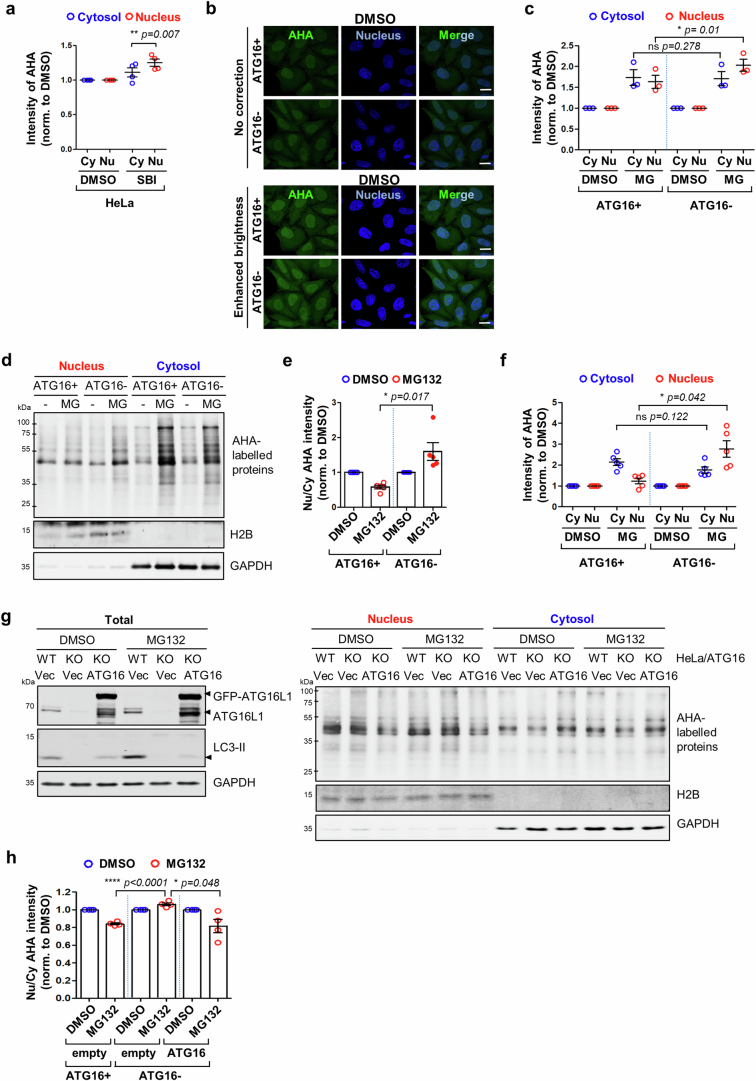
Bulk proteins overflow into nucleus under the autophagy depletion. **a**, Localization of newly synthesized proteins (AHA-labelled proteins) in *ATG16L1* WT (ATG16+) and KO (ATG16-) cells treated with autophagy kinase inhibitor SBI-0206965 (SBI, 5 µM) assessed by Click-iT assay for immunostaining. Data showing a nucleus/cytosol intensity ratio of AHA-labelled proteins in Fig. 2i is divided between AHA-labelled protein intensity in nucleus (Nu, red) and cytosol (Cy, blue) upon SBI treatment. Values are mean ± S.E.M. (n = 4 biologically independent experiments; ** p < 0.01 vs. DMSO; two-tailed paired t-test) **b, c**, Localization of newly synthesized proteins (AHA-labelled proteins) in *ATG16L1* WT (ATG16+) and KO (ATG16-) cells treated with MG132 (MG) using immunofluorescence. **b**, Control data for AHA labelling in *ATG16L1* WT and KO cells in DMSO by immunofluorescence. MG132 data are in Fig. 2j. Upper panel (No correction) shows the raw image micrographs at exposures that match Fig. 2j, for which this is the control. Lower panel (Brightness enhancement) shows increased brightness so that AHA signal can be more easily seen. Scale bar, 20 µm. **c**, Data from the nucleus/cytosol intensity ratio of AHA-labelled proteins in Fig. 2j is divided between AHA-labelled protein intensity in nucleus (Nu, red) and cytosol (Cy, blue) upon MG132 treatment. Values are mean ± S.E.M. (n = 3 biologically independent experiments; ns = not significant; * p < 0.05 for relative changes induced by MG in WT vs. KO cells; two-tailed paired t-test). **d-f**, Localization of newly synthesized proteins (AHA-labelled proteins) in *ATG16L1* WT (ATG16+) and KO (ATG16-) cells treated with MG132 (MG) using immunoblotting after nucleus and cytosol fractionation. **e**, Quantified data from the intensity ratio of nucleus/cytosol-localized AHA-labelled proteins upon MG132 treatment. Values are mean ± S.E.M. Blots (**d**) are representative of 5 biologically independent experiments. (* p < 0.05 for relative changes induced by MG in WT vs. KO cells; two-tailed paired t-test). **f**, Data from the nucleus/cytosol intensity ratio of AHA-labelled proteins in Extended Data Fig. 6e is divided between AHA-labelled protein intensity in nucleus (Nu, red) and cytosol (Cy, blue) upon MG132 treatment. Values are mean ± S.E.M. (n = 5 biologically independent experiments; ns = not significant; * p < 0.05 for relative changes induced by MG in WT vs. KO cells; two-tailed paired t-test). **g, h**, Effect of autophagy rescue by ATG16L1 on newly synthesized protein localization in *ATG16L1* KO cells treated with MG132. **g**, Representative blots show ATG16L1 rescue on autophagy in *ATG16L1* KO (left) and localization of newly synthesized proteins in nucleus or cytosol under the ATG16L1 rescued condition (right) described in Fig. 2k. **h**, Intensity of Nucleus/Cytosol-localized AHA-labelled proteins by immunofluorescence. Values are mean ± S.E.M. (n = 4 independent experiments; **** p < 0.0001 for relative changes induced by MG132 in WT/empty vs. KO/empty cells, * p < 0.05 for relative changes induced by MG132 in KO/empty vs. KO/GFP-ATG16 overexpression (ATG16); two-tailed paired t-test). Source numerical data and unprocessed blots are available in source data. Source data

Based on our findings, we hypothesized that ‘bulk autophagy substrates’, the general pool of cytoplasmic proteins degraded by autophagy, will move to the nucleus in autophagy-inhibited cells. To assess if the transport of newly synthesized proteins into the nucleus was increased in autophagy-null cells, we used two approaches. First, we assessed the localization of newly synthesized misfolded peptides/proteins by treating cells for 2 h with puromycin, an antibiotic that resembles the 3′ end of aminoacylated tRNA (aa-tRNA), which causes premature translational termination (a cytoplasmic event)^25^. *ATG16L1*-null cells had more puromycin-induced misfolded protein inclusions in the nucleus compared with their WT counterparts (Extended Data Fig. 5). Second, we labelled newly synthesized proteins with AHA (l-azidohomoalanine) using ‘click’ chemistry^26,27^ and showed that autophagy inhibition by SBI-0206965 (SBI) increased the intensity of nuclear-localized AHA-labelled proteins (Fig. 2i and Extended Data Fig. 6a). Note that a 10% change in bulk protein in the nucleus versus the cytoplasm is very meaningful from a biological perspective, as many proteins in the cytoplasm (for example, those associated with membranes and organelles) cannot enter the nucleus. More AHA-labelled proteins were localized in the nucleus upon MG132 treatment in *ATG16L1*-null cells compared with WT cells (Fig. 2j and Extended Data Fig. 6b–f). Significantly, we show both by immunoblotting of fractionated cells (Fig. 2k and Extended Data Fig. 6g) and by microscopy (Extended Data Fig. 6h) that reinstatement of autophagy by complementation of *ATG16L1*-null cells with GFP*-ATG16L1* rescued the nuclear/cytoplasmic accumulation of AHA-labelled proteins to normal ratios in the absence or presence of MG132. These data indicate that autophagy inhibition causes excessive translocation of numerous newly synthesized and misfolded proteins into the nucleus (Fig. 2l).

**Fig. 3:**
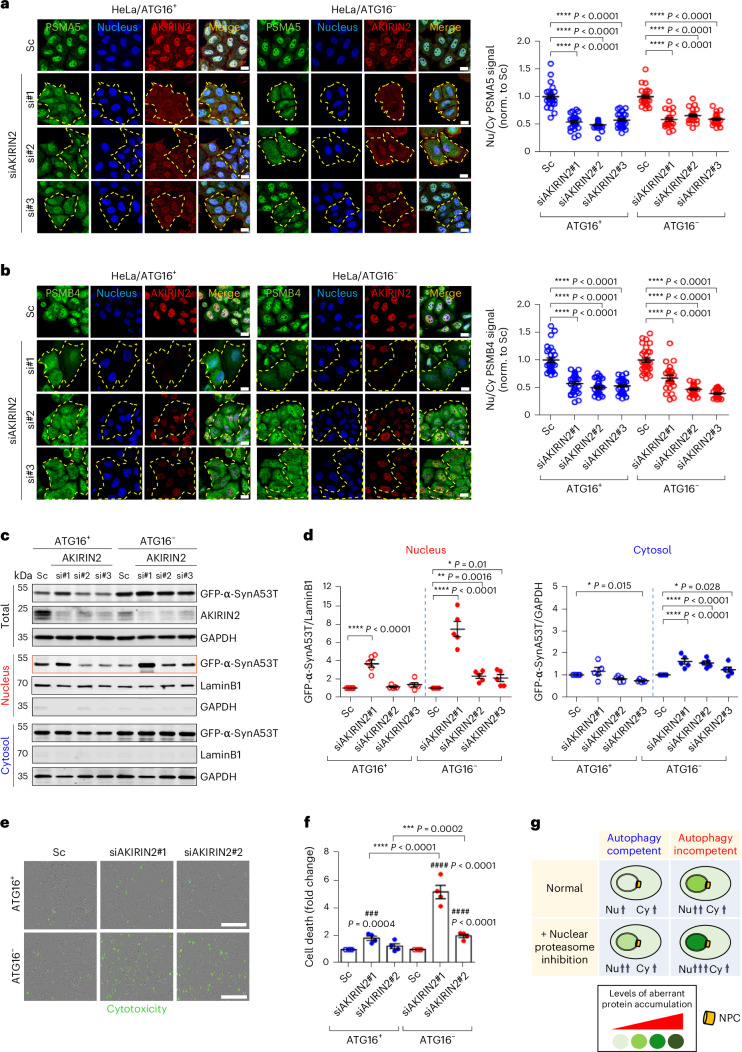
Loss of AKIRIN2 decreases nuclear proteasome-mediated protein degradation in autophagy-deficient cells. **a**,**b**, Knockdown of AKIRIN2 with distinct siRNAs inhibits nuclear localization of proteasome subunits PSMA5 (**a**) and PSMB4 (**b**). Cells were transfected with *AKIRIN2* siRNAs (#1, #2 and #3) for 5 days in *ATG16L1* WT and -null cells. Cells were fixed and labelled for endogenous AKIRIN (red), DAPI (nucleus, blue) and either PSMA5 (green, **a**) or PSMB4 (green, **b**). Quantification of nucleus/cytosol-localized proteasome subunit (PSMA5 (**a**), PSMB4 (**b**) (~*n* = 30 cells in each condition with three different siRNA oligonucleotides; ****P < 0.0001 versus DMSO; one-way ANOVA with post hoc Dunnett test) – single experiment to validate published results^28^ (right). Yellow dashes in **a** and **b** outline AKIRIN2-knockdown cells. Scale bar, 20 μm. **c**,**d**, Knockdown of AKIRIN2 with distinct siRNAs (si#1, si#2 and si#3) inhibits A53T α-Syn degradation in the nucleus assessed by cell fractionation (day 3). Nuclear A53T α-Syn accumulated in *ATG16L1* KO (ATG16^−^) cells after AKIRIN2 knockdown (**c**). Relative changes of GFP-A53T α-Syn levels in nucleus and cytosol within each cell line after AKIRIN2 knockdown (scramble (Sc) = 1) (*n* = 5 independent experiments; *P < 0.05, **P < 0.01, ****P < 0.0001 versus Sc; one-way ANOVA with post hoc Dunnett test) (**d**). **e**, Knockdown of AKIRIN2 enhances cytotoxicity (green fluorescence) in *ATG16L1* KO cells measured by Incucyte live-cell imaging. Scale bar, 600 μm. **f**, Quantification of CellTox Green fluorescence intensity after knockdown of AKIRIN2 (day 5) (*n* = 4 independent experiments; ###*P* < 0.001, ####*P* < 0.0001 versus Sc; ***P < 0.001, ****P < 0.0001 for relative changes induced by specific siRNA in WT versus KO cells; two-way ANOVA with post hoc Tukey test). **g**, Schematic representation shows idealized protein levels/localization upon nuclear proteasome inhibition in WT and autophagy-null cells. Data used for two-way ANOVA derived control values as the sum of all data from that experiment, where controls were originally normalized to 1 (as shown in the graphs), divided by the number of conditions analysed (see  Methods). Data analysed by one-way ANOVA, where ATG16^+^ and ATG16^−^ data were not compared, were analysed separately. Values are mean ± s.e.m. Source numerical data and unprocessed blots are available in source data. DAPI, 4,6-diamidino-2-phenylindole. Source data

Moreover, to confirm that nuclear proteasomes were involved in the degradation of the proteins translocated to this compartment after autophagy inhibition, we knocked down AKIRIN2, a key mediator of proteasome import into the nucleus^28^ (which we replicated in Fig. 3a,b). Loss of AKIRIN2 increased A53T α-Syn levels in the nucleus more in *ATG16L1*-null cells than in WT cells (Fig. 3c,d) and resulted in more cell death in the autophagy-null versus WT cells (Fig. 3e,f). These data suggest that autophagy loss causes proteins to shuttle into the nucleus for degradation by nuclear proteasomes (Fig. 3g).

**Extended Data Fig. 7:**
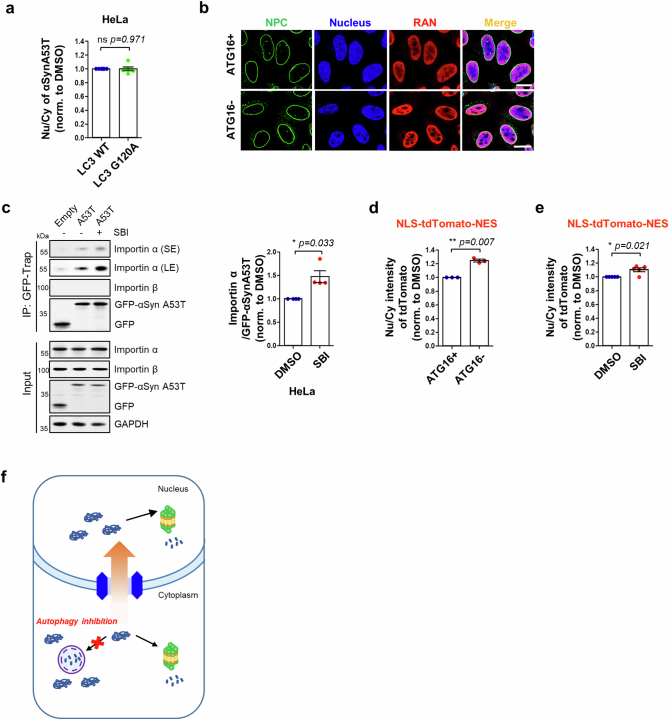
Autophagy compromise causes bulk proteins ‘overflow’. **a**, Quantification of the ratio of nucleus/cytosol-localized αSyn A53T upon either LC3 wild-type (LC3 WT) or non-lipidated form (LC3 G120A) overexpression, by immunostaining. HeLa cells were transfected pHM6-αSyn A53T (HA tag) with either GFP-LC3 wild-type (WT) or GFP-LC3 G120A mutant and then fixed for immunostaining using HA antibody to confirm the localization of αSyn A53T (Value are mean ± S.E.M, n = 6 biological independent experiments; ns = not significant vs. LC3 WT; two-tailed paired t-test). **b**, HeLa/*ATG16L1* wild-type and null cells were fixed and labelled for NPC (nuclear pore complex, green), RAN (Ras-related nuclear protein, red), and DAPI (nucleus, blue). This experiment was performed once simply to confirm an intact NPC in *ATG16L1* WT and KO cells. Scale bar, 20 μm. **c**, Representative blots showing the binding of αSyn A53T with importin α upon autophagy inhibition (SBI, 5 uM) in HeLa cells. Cells were transfected with either GFP-empty or GFP-αSyn A53T and then cells were treated with SBI. Then, immunoprecipitates obtained using GFP-trap were processed for immunoblotting to detect importin α and importin β. (Right graph) Quantification shows the amount of importin α bound GFP-αSyn A53T (Value are mean ± S.E.M; n = 4 biological independent experiments; * p < 0.05 vs. DMSO; two-tailed paired t-test). **d** and **e**, Cells were transfected with NLS–tdTomato-NES which is a shuttling reporter containing both an NLS and an NES fused to tdTomato. **d**, Quantification of a ratio of nucleus/cytosol-localized tdTomato intensity in HeLa/*ATG16L1* wild-type (ATG16+) and null cells (ATG16-) (Value are mean ± S.E.M, n = 3 independent experiments; ** p < 0.01 vs. ATG16 + ; two-tailed paired t-test). **e**, Quantification of a ratio of nucleus/cytosol-localized tdTomato intensity upon SBI treatment for 15 h in HeLa cells. (Value are mean ± S.E.M, n = 5 biological independent experiments; * p < 0.05 vs. DMSO; two-tailed paired t-test) **f**, Schematic representation shows autophagy compromise leads to an “overflow” of bulk proteins (likely autophagy substrates) into the nucleus for degradation by nuclear proteasomes. Source numerical data and unprocessed blots are available in source data. Source data

As ATG16L1 functions in the conjugation of LC3 to phosphatidylethanolamine (PE), resulting in lipidated LC3 (LC3-II)^9^, we assessed whether the non-lipidated form of LC3/ATG8, which would accumulate in autophagy-deficient cells, impacted the cytoplasm-to-nuclear shuttling autophagy substrates in autophagy-null conditions. The non-lipidated LC3 mutant, LC3 G120A, where glycine 120 is replaced with alanine (LC3 G120A)^9^ did not affect the localization of α-Syn A53T, compared with wild-type (WT) LC3 in HeLa cells (Extended Data Fig. 7a).

Furthermore, the cytoplasm-to-nuclear shuttling of proteins in autophagy-null cells is unlikely to be due to aberrant NPCs, as NPC morphology and RAN (Ras-related nuclear protein) localization in autophagy-depleted cells (*ATG16L1* KO) were similar to WT cells (Extended Data Fig. 7b).

We next examined whether autophagy inhibition induces the association of autophagy substrates, like α-Syn A53T, with importins^21^. α-Syn A53T binding with importin α was not significantly affected in autophagy-deficient cells (*ATG16L1* KO) compared with WT (Extended Data Fig. 4k). However, as we compute the strength of binding by dividing the amount of immunoprecipitated material by the amount of immunoprecipitated α-Syn, the system may be oversaturated with α-Syn that accumulates and is immunoprecipitated more abundantly in the autophagy-null cells. To bypass this uncertainty, we acutely inhibited autophagy with the autophagy kinase ULK1/2 inhibitor (SBI) and observed that it caused more α-Syn A53T to interact with importin α (Extended Data Fig. 7c), indicating that some autophagy substrates may have increased binding to importins when autophagy is impaired.

To directly investigate the status of cytoplasm-to-nuclear transport, *ATG16L1* WT and knockout cells were transfected with NLS–tdTomato–NES (Lentiviral-S-tdTomato), which is a shuttling reporter containing both an NLS and an NES fused to tdTomato^29,30^. Autophagy compromise induced either by ATG16L1 depletion or autophagy kinase ULK1/2 inhibition (SBI) resulted in a greater nuclear occupancy of the reporter, suggesting increased cytoplasm-to-nucleus trafficking (Extended Data Fig. 7d,e). Together these data suggest that autophagy compromise causes an ‘overflow’ of bulk cytoplasmic proteins (likely autophagy substrates) into the nucleus (Extended Data Fig. 7f).

### Nucleocytoplasmic shuttling defect in Huntington’s disease

**Fig. 4:**
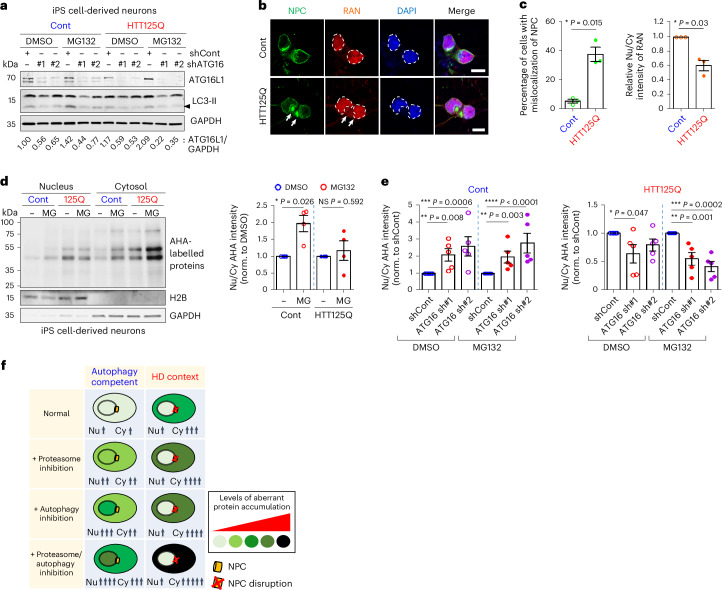
Cytoplasm-to-nucleus shuttling of bulk proteins is inhibited in Huntington’s disease cells. **a**, Inhibition of autophagy using distinct shRNA targeting ATG16L1 (#1 and #2) decreases LC3-II and ATG16L1 levels in control (Cont) and iPS cell-derived neurons from a juvenile patient with HD originally carrying 125 CAGs (HTT125Q) with or without MG132 (MG, 1 µM for 15 h). Representative blot of three biological repeats. **b**,**c**, NPC disruption in HTT125Q-derived neurons was detected by staining of either NPC or RAN, compared with Cont iPS cell-derived neurons (**b**). Arrows indicate the signals of NPC or RAN in the cytoplasm. Scale bar, 10 μm. Quantified data represents mislocalization of NPC (left) or RAN (right) in HTT125Q-derived neurons (**c**). Values are mean ± s.e.m. (*n* = 3 independent experiments; *P < 0.05 versus Cont; two-tailed paired *t*-test). White dashes in **b** indicate nuclear outline. **d**, Decreased amount of nuclear-localized newly synthesized proteins (AHA-labelled proteins) in HTT125Q-derived neurons upon MG132 (2 µM for 15 h) treatment, compared with Cont iPS cell-derived neurons. Values are mean ± s.e.m. (*n* = 4 independent experiments; NS, not significant; *P < 0.05 versus DMSO; two-tailed paired *t*-test). **e**, Autophagy compromise exacerbated mislocalization of newly synthesized proteins in cytosol upon MG132 (1 µM for 15 h) treatment in HTT125Q-derived neurons (right) compared to Cont iPS cell-derived neurons (left). Values are mean ± s.e.m. (*n* = 5 independent experiments; *P < 0.05, **P < 0.01, ***P < 0.001, ****P < 0.0001 versus shCont; one-way ANOVA with post hoc Dunnett test). Data used for one-way ANOVA, where DMSO and MG132 data were not compared, were analysed separately. **f**, Schematic representation shows idealized protein localization/abundance upon proteasome inhibition, autophagy inhibition or combined proteasome/autophagy inhibition in the HD context. Source numerical data and unprocessed blots are available in source data. Source data

**Extended Data Fig. 8:**
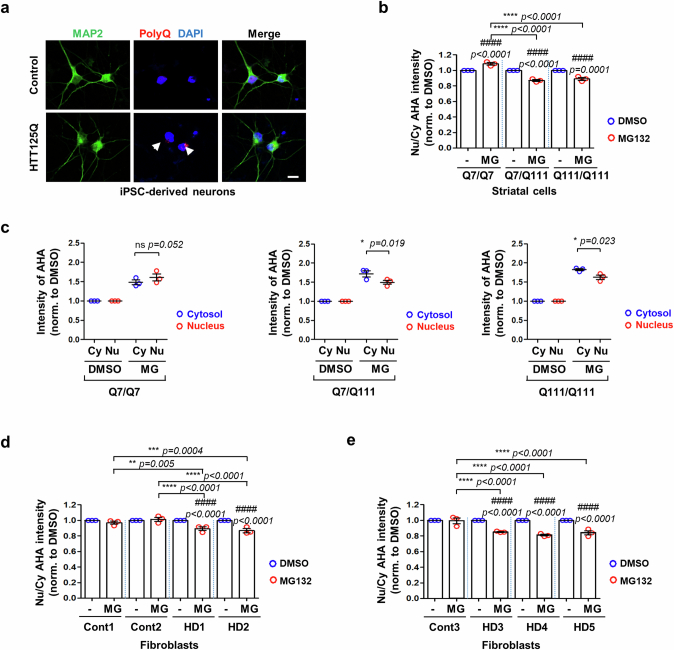
Relocation of newly synthesized proteins to the nucleus is inhibited in HD upon proteasome inhibition. **a**, Validation of HTT125Q-derived neurons by immunofluorescence compared to control iPSC-derived neurons using polyQ antibody and MAP2 antibody (neuronal marker). Arrowhead indicates huntingtin aggregates. Scale bar, 10 μm. **b** and **c**, Reduced nuclear localization of newly synthesized proteins (AHA-labelled proteins) in mouse striatal cells expressing homozygous mutant (Q111/Q111) or heterozygous mutant (Q7/Q111) huntingtin upon MG132 treatment (MG, 1 µM for 15 h) compared with Q7/Q7 (wild-type). **b**, Quantified data from the intensity ratio of nucleus/cytosol-localized AHA-labelled proteins upon MG132 treatment. Values are mean ± S.E.M. (n = 3 independent experiments; #### p < 0.0001 vs. DMSO; **** p < 0.0001 for relative changes induced by MG132 in wild-type vs. HD striatal cells; two-way ANOVA with post hoc Tukey test). **c**, Data from the nucleus/cytosol intensity ratio of AHA-labelled proteins in (**b**) is divided between AHA-labelled protein intensity in nucleus (Nu, red) and cytosol (Cy, blue) upon MG132 treatment. Values are mean ± S.E.M. (n = 3 independent experiments; ns = not significant; * p < 0.05 for relative changes induced by MG132 in cytosol vs. nucleus. **d**, Localization of newly synthesized proteins in HD fibroblasts expressing mHTT-polyQ65 (HD1) or mHTT-polyQ80 (HD2) compared with control (Cont1, Cont2). Decreased nuclear localization of newly synthesized proteins in HD fibroblasts (HD1, HD2) compared with Controls (Cont1, Cont2) treated with MG132 (1 µM for 15 h). Values are mean ± S.E.M. (n = 3 independent experiments; #### p < 0.0001 vs. DMSO; ** p < 0.01,*** p < 0.001, **** p < 0.0001 for relative changes induced by MG132 in Cont vs. HD fibroblasts; two-way ANOVA with post hoc Tukey test). **e**, Localization of newly synthesized proteins in HD fibroblast (HD3, HD4, HD5) compared with control (Cont3). Decreased nuclear localization of newly synthesized proteins in HD fibroblasts compared with control (Cont3) upon MG132 treatment (1 µM for 15 h). Values are mean ± S.E.M. (n = 3 independent experiments; #### p < 0.0001 vs. DMSO; **** p < 0.0001 for relative changes induced by MG132 in Cont vs. HD fibroblasts; two-way ANOVA with post hoc Tukey test). Source numerical data are available in source data. Source data

HD is an autosomal dominant neurodegenerative disorder which is associated with mild autophagy inhibition and disruption of the NPC and nucleocytoplasmic transport^1,31^. Consistent with previous data in various systems in cell culture and in vivo^3,26,32^, induced pluripotent stem (iPS) cell-derived neurons from a juvenile patient with HD originally carrying 125 CAG trinucleotide repeats (HTT125Q) (Extended Data Fig. 8a)^33^ had lower levels of LC3-II/autophagosomes than controls (Fig. 4a)^32^. In line with recent studies suggesting that HD is associated with defective nucleus-cytoplasmic transport^31^, HTT125Q-derived neurons had compromised nuclear membrane integrity, and mislocalization of the NPC and RAN (which enables translocation of proteins and RNA through the NPC) to the cytosol, compared with control (Fig. 4b,c).

As modest autophagy compromise is likely quite well tolerated in humans^34^, we considered whether cells with the HD mutation would manifest the biochemical alterations that would be predicted from a combined deficiency in autophagy and cytoplasm-to-nuclear transport. Accordingly, we tested whether the normal ability of cells to cope with autophagy compromise by shuttling cytoplasmic substrates to the nucleus for proteasomal degradation was compromised in cells carrying the HD mutation. A lower abundance of newly synthesized bulk proteins was present in the nucleus, in HTT125Q iPS cell-derived neurons compared with controls (Fig. 4d), in mouse striatal cells with homozygous (Q111/111) and heterozygous (Q7/Q111) knock-ins of the HD mutation compared with WT cells (Q7/Q7) (Extended Data Fig. 8b,c) and in HD patient fibroblasts compared with controls (Extended Data Fig. 8d,e), suggesting impaired cytoplasm-nuclear transport. These experiments were conducted in cells treated with proteasome inhibitors to limit degradation of the nuclear pool to enable complete ascertainment of the nuclear transport of newly synthesized proteins in the cytoplasm.

**Extended Data Fig. 9:**
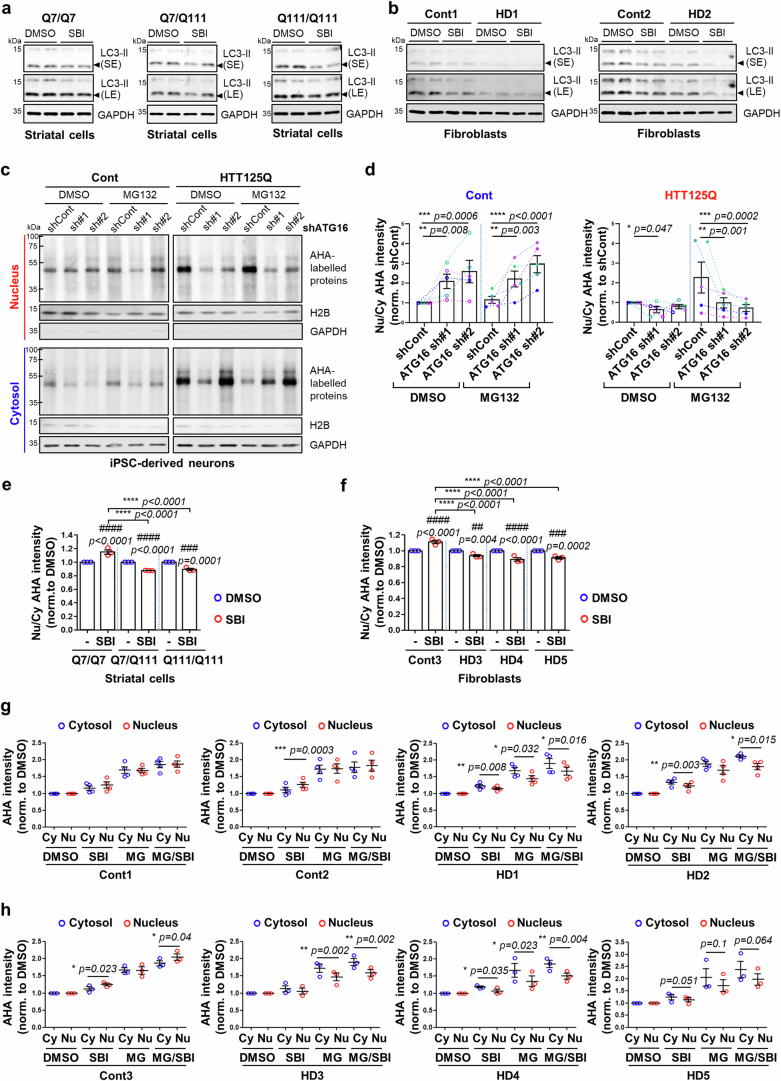
Cytoplasm to nucleus trafficking of AHA-labelled proteins is inhibited in HD in response to both autophagy and proteasome inhibition, leading to detrimental consequences. **a**, Confirmation of autophagy inhibition (LC3-II) by SBI (5 µM for 15 h) in mouse striatal cells expressing wild-type (Q7/Q7), mutant (Q111/Q111) or heterozygous (Q7/Q111). **b**, Confirmation of autophagy inhibition (LC3-II) by SBI treatment (10 µM for 15 h) in HD fibroblasts (HD1, HD2) compared to control (Cont1, Cont2). **a** and **b** are experiments performed once to validate behaviour of SBI compound that has been used extensively in the literatures and in the lab in other contexts. **c** and **d**, Autophagy compromise exacerbates mislocalization of newly synthesized proteins (AHA-labelled proteins) in cytosol upon MG132. Control iPSC-derived neurons and HTT125Q-derived neurons were infected with shCont or shATG16 (sh#1, sh#2). After 4 days, neurons were treated with MG132 (1 µM) for 15 h. Effect of autophagy compromise (shATG16 (sh#1, sh#2)) on mislocalised newly synthesized proteins (AHA-labelled proteins) in HTT125Q-derived neuron with or without MG132 by immunoblotting described in Fig. 4e. Raw data from Fig. 4e quantified data is shown in **d**. In Fig. 4e, the graphs show the ratio of nucleus-localized AHA-labelled proteins/cytosol-localized AHA-labelled proteins (Nu/Cy AHA intensity) normalized to shCont in each condition (either DMSO (1^st^ lane of the graph) or MG132 treatment (4^th^ lane of the graph), respectively). (**d**) The graphs show the ratio of Nu/Cy AHA intensity was normalized to shCont with DMSO treatment (1^st^ Lane) in Control iPSC-derived neurons (left) and HTT125Q-derived neurons (right). Each dot with same colour indicates the same biological repeat experiment. (Value are mean ± S.E.M, n = 5 biologically independent experiments; * p < 0.05, ** p < 0.01, *** p < 0.001, **** p < 0.0001 vs. shCont; one-way ANOVA with post hoc Dunnett test) Data used for one-way ANOVA where DMSO and MG132 were not compared and we analysed separately shown in Fig. 4e. **e**, Autophagy inhibition (SBI 5 µM for 15 h) decreases newly synthesized proteins in nucleus compared to the cytosol in mouse striatal cells expressing Q111/Q111 and Q7/Q111 versus Q7/Q7 (wild-type). We did these experiments at the same time as Extended Data Fig. 8b,c and these shared the same untreated/DMSO treated condition. Values are mean ± S.E.M. (n = 3 independent experiments; ### p < 0.001, #### p < 0.0001 vs. DMSO; **** p < 0.0001 for relative changes induced by SBI in wild-type vs. HD striatal cells; two-way ANOVA with post hoc Tukey test). **f**, Autophagy inhibition (SBI 10 µM for 15 h) inhibits nuclear localization of newly synthesized proteins in HD fibroblasts (HD3, HD4, HD5) compared to control (Cont3). Values are mean ± S.E.M. (n = 3 independent experiments; ## p < 0.01, ### p < 0.001, #### p < 0.0001 vs. DMSO; **** p < 0.0001 for relative changes induced by SBI in Cont vs. HD fibroblasts; two-way ANOVA with post hoc Tukey test). **g, h**, Nucleus (red) and cytosol (blue) localized newly synthesized proteins in response to autophagy inhibitor (SBI, 10 µM for 15 h) and/or proteasome inhibitor (MG132 1 µM for 15 h) in Cont (Cont1, Cont2, Cont3) and HD fibroblasts (HD1, HD2, HD3, HD4, HD5). Decreased nuclear-localized newly synthesized proteins in HD fibroblasts versus control upon autophagy inhibition and/or proteasome inhibition. Values are mean ± S.E.M. (Cont1 (n = 4 independent experiments), Cont2 (n = 4 independent experiments), Cont3 (n = 3 independent experiments), HD1 (n = 4 independent experiments), HD2 (n = 4 independent experiments), HD3 (n = 3 independent experiments), HD4 (n = 3 independent experiments), HD5 (n = 3 independent experiments); * p < 0.05,** p < 0.01,*** p < 0.001 for nucleus vs. cytosol intensity; two-tailed paired t-test). Data in (Extended Data Figs. 8e and 9f) are divided between the AHA-labelled protein intensity in nucleus and cytosol in the condition of proteasome inhibition (Extended Data Fig. 8e), autophagy inhibition (Extended Data Fig. 9f) and both in (**h**) for ease of viewing. Source numerical data and unprocessed blots are available in source data. Source data

Autophagy inhibition with *ATG16L1* knockdown in WT iPS cell-derived neurons (Fig. 4a) or with SBI-0206965 in WT striatal cells (Extended Data Fig. 9a) and WT fibroblasts (Extended Data Fig. 9b) increased nuclear levels of newly synthesized proteins (with or without proteasome inhibition), consistent with our model that cells cope with the accumulation of cytoplasmic autophagy substrates by trafficking these to the nucleus for proteasomal degradation (Extended Data Fig. 9c–f). However, in HD iPS cell-derived neurons (Fig. 4e and Extended Data Fig. 9c,d), HD mutant striatal cells (Extended Data Fig. 9e) and HD fibroblasts (Extended Data Fig. 9f–h), the levels of newly synthesized proteins in the nucleus were reduced after autophagy inhibition, suggesting that HD cells do not have efficient means of relocating the accumulating autophagy substrates proteins from the cytoplasm to the nucleus (Fig. 4f).

**Extended Data Fig. 10:**
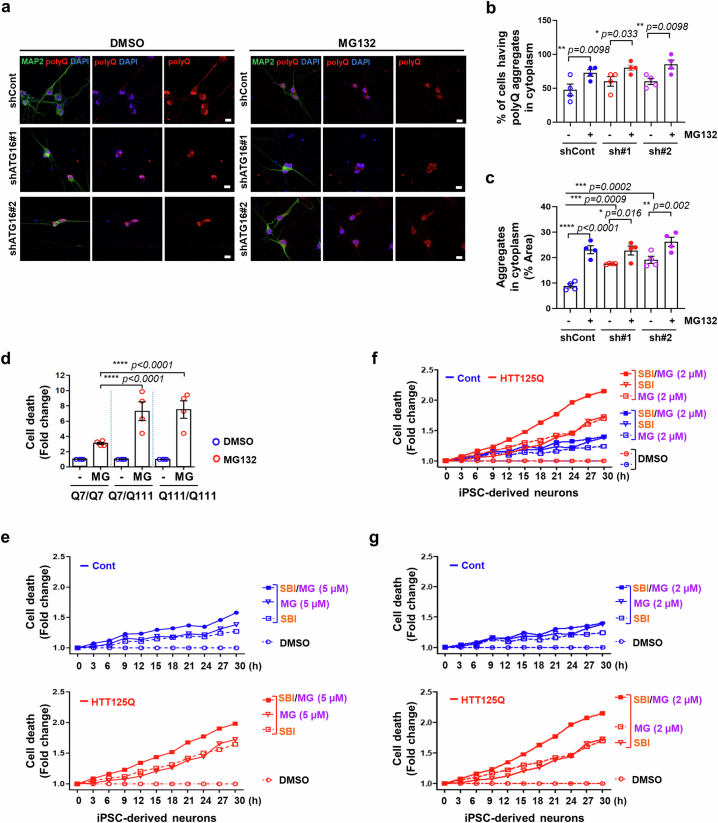
Cytoplasm to nucleus trafficking of AHA-labelled proteins is inhibited in HD in response to both autophagy and proteasome inhibition, leading to detrimental consequences. **a-c**, HTT125Q-derived neurons were infected with either shControl (shCont) or shATG16 (sh#1, sh#2) for 4 days and then neurons were treated with either DMSO or MG132 for 15 h. **a**, Neurons were fixed and labelled with PolyQ (red), neuronal marker MAP2 (green) and DAPI (blue). Scale bar, 10 μm. **b**, Quantification of the percentage of cells having polyQ aggregates in cytoplasm of the cell body (Value are mean ± S.E.M, n = 4 biological independent experiments; * p < 0.05, ** p < 0.01 vs. DMSO; two-way ANOVA with post hoc Tukey test). **c**, Quantification of the percentage of cytoplasmic area in the cell body occupied by aggregates upon autophagy compromise and/or proteasome inhibition (Value are mean ± S.E.M, n = 4 biological independent experiments; * p < 0.05, ** p < 0.01, *** p < 0.001, **** p < 0.0001 vs. DMSO; two-way ANOVA with post hoc Tukey test). **d**, The effect of prolonged proteasome inhibition on cell death in HD mouse striatal cells. Cell death was detected by CellTox Green after MG132 treatment (1 µM) for 24 h. (Value are mean ± S.E.M, n = 4 biological independent experiments; **** p < 0.0001 for relative changes induced by MG132 in Cont vs. HD striatal cells; two-way ANOVA with post hoc Tukey test) **e-g**, Effect of autophagy inhibition (SBI) and/or proteasome inhibition (MG) on cell death in control iPSC-derived neurons and HTT125Q-derived neurons. Cell death measured CellTox Green fluorescence by Incucyte live-cell imaging in a time-dependent manner. The cell death experiments in Fig. 5a are shown with a high concentration of MG132 (Fig. 5a, Extended Data Fig. 10e (Cont and HTT125Q, respectively)) and the same experiment with a low concentration of MG132 is shown in (**f** and **g**) for ease of viewing. **e**, Representative graph with three biologically independent experiments described in Fig. 5a. Cell death by autophagy inhibition (SBI, 10 µM) and/or proteasome inhibition (MG, 5 µM) in control iPSC-derived neurons (top) and HTT125Q-derived neurons (bottom). Data in Fig. 5a are divided between control and HTT125Q-derived neurons in (**e**) for ease of viewing. **f, g**, Graphs showing cell death in Control and HTT125Q iPSC-derived neurons treated with proteasome inhibitor MG132 (MG, 2 µM), autophagy inhibitor SBI (10 µM) and/or both (SBI + MG). Data in (**g**) show the data extracted from (**f**) restricted to DMSO, SBI, MG and SBI + MG in control iPSC-derived neurons (top) and HTT125Q-derived neurons (bottom) to enable ease of comparisons. These graphs do not show error bars which make the graphs too messy. The raw data is shown in the data files with *P* values (n = 3 biologically independent experiments; one-tailed paired t-tests). When we compute areas under the curve for 3 biological replicates, then SBI vs. SBI + MG in Cont iPSC-derived neurons p = 0.032; SBI vs SBI + MG in HTT125Q-derived neurons p = 0.073; SBI in Cont vs. HTT125Q-derived neurons p = 0.078; MG in Cont vs. HTT125Q-derived neurons p = 0.025; SBI + MG in Cont vs. HTT125Q-derived neurons p = 0.022 (one-tailed paired t-tests). Autophagy inhibition plus proteasome inhibition causes more cell death than each inhibition alone in HTT125Q-derived neurons compared to control iPSC-derived neurons. Source numerical data are available in source data. Source data

Notably, in HD neurons with and without autophagy or proteasome inhibition, aggregates are predominantly cytoplasmic and can be seen in both the cell body and neurites. It was difficult to determine whether nuclei had aggregates or more intense huntingtin staining. Autophagy inhibition increased the cytoplasmic area occupied by aggregates; in contrast, proteasome inhibition increased the percentage of cells with aggregates in the cytoplasm, in the cell body and their area/volume (Extended Data Fig. 10a–c). There may be ceiling effects with these observations as there will likely be a maximum volume of the cells occupied by aggregates that can be tolerated before cell death occurs.

**Fig. 5:**
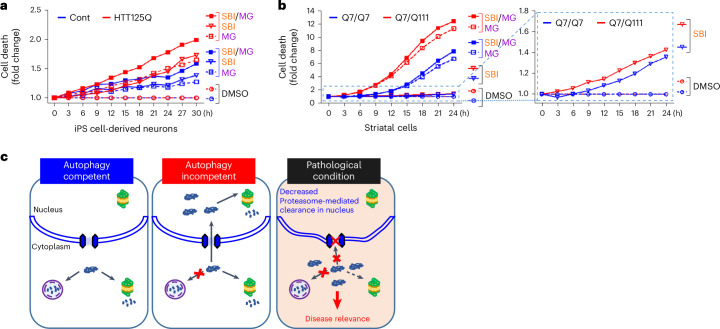
Defects in cytoplasm-to-nucleus shuttling of bulk proteins exacerbate cell death in Huntington’s disease cells. **a**, Real-time CellTox Green fluorescence as a result of cell death is monitored by Incucyte in a time-dependent manner. Exacerbated cell death by combined inhibition of autophagy and proteasome (SBI and MG, respectively) in HTT125Q-derived neurons. These graphs do not show error bars, which make the graphs less clear. The raw data are shown in the data files with *P* values. When we compute areas under the curve for three biological replicates, then SBI versus SBI + MG in Cont iPS cell-derived neurons *P* = 0.029; SBI versus SBI + MG in HTT125Q-derived neurons *P* = 0.049; SBI in Cont versus HTT125Q-derived neurons *P* = 0.078; MG in Cont versus HTT125Q-derived neurons *P* = 0.036; SBI + MG in Cont versus HTT125Q-derived neurons *P* = 0.016 (one-tailed paired *t*-test). **b**, Left graph: enhanced cell death caused by combined inhibition of autophagy and proteasome in mouse striatal cells expressing Q7/Q111 compared with each inhibitor alone. Right graph: increased cell death caused by autophagy inhibition (SBI) in Q7/Q111 compared with Q7/Q7 striatal cells (expanded scale from the same data in the left-hand graph to clarify effects of SBI to enable ease of comparison). When we compute areas under the curve for four biological replicates normalized to DMSO values showing Q7/Q7 and Q7/Q111 striatal cells treated with proteasome inhibitor MG132 (MG, 2 µM), autophagy inhibitor SBI (5 µM) and/or both (SBI + MG) in a time-dependent manner, then SBI versus SBI + MG (in Q7/Q7 cells): *P* = 0.036, SBI versus SBI + MG (in Q7/Q111 cells): *P* = 0.015, SBI in Q7/Q7 versus Q7/Q111: *P* = 0.274, MG in Q7/Q7 versus Q7/Q111: *P* = 0.024, SBI + MG in Q7/Q7 versus Q7/Q111: *P* = 0.029 (one-tailed paired *t*-test). **c**, Schematic summary for our study. Cytoplasmic substrates accumulating during autophagy depletion move into the nucleus via nuclear pores to be degraded by nuclear proteasomes. In the HD context, defective cytoplasm-to-nuclear transport enhances susceptibility to autophagy inhibition leading to cell dysfunction. Source numerical data are available in source data. Source data

As the HD cells already have a degree of autophagy compromise, this suggests that they are prone to synergistic viability changes akin to synthetic fitness loss due to impaired autophagy and compromised cytoplasm-to-nuclear transport reducing the ability of the cells to manage to ‘overflow’ of cytoplasmic autophagic substrates by nuclear proteasomal degradation. Indeed, MG132 caused more cell death in both iPS cell-derived neurons (Fig. 5a) and striatal cells with the HD mutation compared with controls (Fig. 5b and Extended Data Fig. 10d), consistent with their autophagy defect. Furthermore, HD disease cells were also more sensitive to autophagy inhibition and joint autophagy and proteasome inhibition (Fig. 5a,b and Extended Data Fig. 10e–g). This is consistent with our model that the defective cytoplasm-to-nuclear transport in HD mutation-carrying cells also makes them more vulnerable to autophagy inhibition – two synergistic effects compromising fitness that are inherently present in these cells to a mild extent (Figs. 4f and 5c).

## Discussion

In conclusion, our data reveal that the synthetic lethality of autophagy gene mutants with those encoding the nuclear core complex or proteasome components that is seen in yeast is conserved as synergistic loss of viability in human cells. These negative interactions can be attributed to cells responding to the accumulation of cytoplasmic autophagic substrates in autophagy deficiency by trafficking them into the nucleus via the nuclear pores to enable degradation by nuclear proteasomes. The need for nuclear proteasomes in this scenario may reflect the saturation of cytoplasmic proteasomal machinery or the possibility that the nucleolus may enable better protein quality control^35^. Both autophagy and NPC activities are compromised in HD, and we propose that the negative genetic interaction resulting from the loss of these processes may be a much more potent driver of pathology than the multiplicative effects of both modest defects. The negative genetic interaction between defective autophagy and compromised nuclear pore function is not confined to HD, as both defects have been reported in other diseases, like motor neuron disease caused by *C9orf72* mutations and tauopathy^36–38^. Thus, we propose that such interactions between different pathways may be critical drivers of many monogenic and complex diseases.

## Methods

These experiments did not require specific ethics approval, except for patient-derived cell lines, which were obtained with appropriate approvals.

### Cell culture

HeLa (human cervical epithelium) (ATCC; CCL-2; CVCL_0030), HEK293FT (human embryonic kidney cell line; Invitrogen, R70007), striatal neuronal cell lines derived from WT HTT Q7/Q7, heterozygous HTT Q7/Q111 and homozygous HTT Q111/Q111 knock-in mice (Coriell Institute CH00097, CH00096 and CH00095, respectively), which are neuronal progenitor cell lines from E14 striatal primordia of HdhQ111 knock-in and WT littermate embryos generated using tsA58 SV40 large T antigen^39^, were maintained in Dulbecco’s modified Eagle’s medium (DMEM) (4.5 mg l^−1^ glucose; Sigma) supplemented with 10% FBS (Sigma-Aldrich, F7524), 2 mM l-glutamine (Sigma-Aldrich, G7513) and 100 U ml^−1^ penicillin-streptomycin (Sigma-Aldrich, P0781). Primary fibroblasts from three unaffected controls (Ctrl, Coriell Institute, GM04711 (Cont1); GM04729 (Cont2), GM04865 (Cont3)), five patients with HD (Coriell Institute GM21757 (HD1), GM0485 (HD3), GM04287 (HD4), GM21756 (HD5); polyQ17/80 HD30501 (HD2) was from F. Squitieri, were cultured in GlutaMAX medium (Gibco) supplemented with 20% FBS (Sigma-Aldrich, F7524), 100 U ml^−1^ penicillin-streptomycin (Sigma-Aldrich, P0781), MEM non-essential amino acid solution (Sigma-Aldrich, M7145) and 2 mM l-glutamine (Sigma-Aldrich, G7513). Ub-G76V-GFP-expressing stable HeLa cell line, which was described previously^26^. Autophagy-deficient ATG16L1 CRISPR KO (ATG16L1 KO) HeLa cells and ATG9 CRISPR KO (ATG9 KO) HeLa cells, together with corresponding autophagy-competent control lines (WT HeLa), were generated by our group following previously published protocols^9^.

All cell lines were incubated at 37 °C (except for striatal neuronal cells maintained at 33 °C) and 5% CO_2_, in a humidified atmosphere and were regularly tested for *Mycoplasma* contamination every 2 weeks. All cell lines were authenticated by the provider company and/or by western blot analysis of specific proteins.

### Human iPS cell culture and iNeuron differentiation

The G3 line of iPS cells previously derived from WTC11 (Cont iPS cells) were kindly provided by M. E. Ward (National Institute of Neurological Disorders and Stroke, National Institutes of Health, Bethesda, Maryland)^40^. For HTT125Q iPS cells, iPS cells were generated from peripheral blood mononuclear cells donated by a juvenile patient with HD originally carrying 125 CAGs (HTT125Q). Stem cells were cultured in E8 Flex medium (Gibco, A2858501) on Vitronectin (Thermo Scientific, A14700)-coated plates at 37 °C, 5% CO_2_. iPS cells were dissociated with 0.5 mM EDTA when reaching 70% confluency for the maintenance. Neuronal differentiation was performed as described previously^33,41^. In brief, iPS cells were dissociated into single cells using accutase and then split to 250,000 cells per well in a Geltrex (Thermo Fisher Scientific, A1413302)-coated six-well plate. On the following day, the medium was changed with the induction medium (DMEM/F12 (Gibco, 21331-020), N2 supplement (100×; Gibco, 17502-048), GlutaMAX (100×; Gibco, 35050-061), non-essential amino acids (100×; Gibco, 11140-035), 2-mercaptoethanol (1,000×; Gibco, 31350-010), penicillin-streptomycin (100×; Gibco, 15140-122 (10,000 U ml^−1^) with doxycycline (1 µg ml^−1^)). For differentiation, cells were switched to Neurobasal medium (NB growth medium) consisting of Neurobasal (Gibco, 21103-049), B27 supplement (50×; Gibco, 17504-044), GlutaMAX (100×; Gibco, 35050-061), 2-mercaptoethanol (1,000×; Gibco, 31350-010), penicillin-streptomycin (100×; Gibco, 15140-122), NT3 (10 ng ml^−1^; Peprotech, 45003), BDNF (10 ng ml^−1^; Peprotech, 45002) with doxycycline (1 µg ml^−1^; Sigma-Aldrich, D9891). On day 4 after induction, cells were plated onto PDL (Gibco, A38904-01)/Geltrex double-coated dishes depending on the experimental purpose. NB growth medium was changed on the following day. After day 7, the cultures were maintained in NB growth medium without doxycycline and changed every other day.

### Cas9 stable cell line

For Cas9 stable cell lines, ATG16L1 WT and KO and ATG9 WT and KO were transduced virus carrying LentiCas9 blast (pKLV-Cas9 blast) in medium supplemented with 6 μg ml^−1^ Polybrene (Sigma-Aldrich, H9268). Infected cells were selected with 10 μg ml^−1^ blasticidin (Sigma-Aldrich, 15205). Cas9 stable cell lines (the pool) were assessed for Cas9 cutting efficiency with a lentiviral vector encoding BFP, GFP and an sgRNA against GFP. The percentage of BFP^+^/GFP^−^ (edited) to BFP^+^/GFP^+^ (total transduced) cells was analysed by FACS to identify Cas9-positive and Cas9-negative cells, respectively, using the LSRFortessa flow cytometer (BD). Data were analysed using FlowJo v.10.7.1 Software (BD Life Sciences)^42,43^.

### Antibodies and reagents

The following primary antibodies were used for western blot and immunofluorescence (IF): rabbit monoclonal anti-LC3B (ab192890), mouse monoclonal anti-GAPDH (ab8245), rabbit polyclonal anti-GFP (ab6556), rabbit polyclonal anti-Lamin B1 (ab16048), rabbit monoclonal anti-Nup98 (ab124980), rabbit monoclonal anti-Nup133 (ab155990), rabbit monoclonal anti-PSMD7 (ab181072), mouse monoclonal anti-NPC proteins antibody (ab24609), mouse monoclonal anti-KPNB1 (3E9) (ab2811); rabbit polyclonal anti-KPNA2 (ab70160) from Abcam; Rabbit polyclonal anti-RAN (10469-1-AP), rabbit polyclonal anti-Lamin B1 (12987-1-AP), rabbit polyclonal anti-H2B (15857-1-AP), mouse monoclonal anti-GFP (66002-1Ig), rabbit polyclonal anti-GFP (50430-2-AP), rabbit polyclonal anti-LC3 (14600-1-AP) from Proteintech; rabbit anti-K48-linkage polyubiquitin (8081), rabbit polyclonal anti-PSMA5 (2457), rabbit monoclonal anti-ATG16L1 (8089) from Cell Signaling; rabbit polyclonal anti-ATG16L1 (MBL, PM040); mouse anti-puromycin (MABE343), mouse anti-polyglutamine-expansion (MAB1574) anti-ubiquitinylated proteins (FK2) (04-263) from Millipore; rabbit polyclonal anti-AKIRIN2 (Atlas Antibodies, HPA064239); mouse monoclonal anti-C6ORF166 (3D9) (AKIRIN2) (Abnova, H00055122-M01); mouse monoclonal anti-PSMB4 (H-3) (Santacruz, sc390878); and mouse monoclonal anti-HA.11 clone 16B12 (Covance, MMS-101P). All primary antibodies were used for overnight incubation at 4 °C and secondary antibodies were used at a dilution of 1:4,000 with incubation for 1 h at room temperature. The secondary antibodies used for IF were conjugated to Alexa Fluor 488 or 594 (Invitrogen). The secondary antibodies used for western blot were the horseradish peroxidase (HRP)-conjugated secondary antibodies, anti-mouse (NA931V, GE Healthcare) and anti-rabbit (NA934V, GE Healthcare) from GE Healthcare and the LI-COR secondary antibodies, anti-mouse 680 and anti-rabbit 800.

The reagents used included MG132 (C2211), SBI-0206965 (SML1540), ivermectin (I8898) and importazole (SML0341) from Sigma-Aldrich; bortezomib (PS-341) (Selleck Chem, S1013), InstantBlue Coomassie Protein Stain (ISB1L) (Abcam, ab119211) and O-propargyl-puromycin (Jena Bioscience, NU-931-05). Reagents were dissolved with DMSO (Sigma-Aldrich). Other reagents were 500 µg ml^−1^ Geneticin Selective Antibiotic (G418, Gibco, 11811-031), MEM amino acids solution (11130-051) and non-essential amino acids solution (11140-050) from Thermo Scientific.

### Plasmids and siRNAs

The pre-designed siRNAs (On-Target Plus SMART pool and/or sets of deconvoluted oligonucleotides) and pre-designed pLKO.1 shRNAs vectors were acquired from the RNAi Consortium are described in Supplementary Table 5.

The following constructs were used: the enhanced GFP (EGFP)-tagged mutant α-synuclein (pEGFP-A53T α-syn) and pHM-mutant α-synuclein (pHM-A53T α-syn) was produced by our group^44^. EGFP-tagged ATG16L1 (pEGFP-ATG16L1)^45^ and EGFP-empty (EGFP-C1) was purchased from Clontech. EGFP-tagged LC3 (LC3 WT) and EGFP-LC3-G120A (LC3 G120A) constructs were kind gifts from T. Yoshimori (Osaka University, Japan)^9^. The LentiCas9 blast vector and the Lenti-PB (pKLV-PB-U6 gRNA(BbsI)-PGKpuro2ABFP) gRNA cloning vector for the CRISPR/Cas9 screen were kindly gifted from E. Metzakopian, UK DRI^42^.

### Transfection

For siRNA transfection, HeLa cells were plated in 6 well plates and transfected with 100 nM siRNA using Lipofectamine RNAi max (Invitrogen, 13778150) for 4–6 h, according to the manufacturer’s instructions. On the following day, cells were split if required and then target genes were knocked down with a second siRNA transfection. To measure cytotoxicity in 96-well plates, HeLa cells were cultured in 96-well plates and then transfected with siRNAs using Lipofectamine 2000 (Invitrogen, 11668) in growth medium without antibiotics for 4–6 h, following the manufacturer’s instructions. Transfected cells were re-seeded in 96-well plates, and followed by another siRNA post-transfection after 48 h. siRNAs were used at 30 nM. After transfections, cells were incubated with full growth medium.

For DNA transfection, cells were seeded in 6 well plates and cells were transfected with 1 µg of DNA with TransIT-2020 reagent (Mirus, MIR5400), following the manufacturer’s instructions. The following day, cells were re-seeded for the relevant experimental requirements.

### Lentivirus production and infection

shRNA lentiviral particles were produced and transduced following the RNAi Consortium protocols as described previously^46^. In brief, HEK293FT packaging cells in 100 mm dishes were co-transfected at 60–70% confluence with a mix of 2.5 μg psPAX2 vector (packaging vector), 270 ng pMD2.G vector (envelope vector) and 2.7 µg hairpin-pLKO.1 vector using TransIT-LT1 (Mirus) transfection reagent according to the manufacturer’s instructions. Transfected cells were cultured in high-serum medium (20% FBS). After 40 h, cell culture medium containing the virus was collected and replaced by high-serum medium at three times repeatedly every 24 h. Viral preps were then concentrated by centrifugation at 160, 100*g* for 90 min. For infection in iPS cell-derived neurons, viruses were added to the iPS cell-derived neurons and incubated overnight. On the following day, medium was replaced by full growth medium and cells were further incubated for an additional 4 days before testing the knockdown effects.

For synthetic lethality (SL) CRISPR/Cas9 arrayed screening, gRNA virus production for 96-well plates was performed as previously described^42,43^. In brief, individual lentiviral plasmids containing an sgRNA were transfected into HEK293FT packaging cells in a 96-well plate format (sgRNA sequences in Supplementary Table 6). HEK293FT packaging cells in 96-well plates were co-transfected at 70% confluence with a mix of 19.5 ng psPAX2 vector (packaging vector), 12.5 ng pMD2.G vector (envelope vector) and 25 ng lentiviral plasmid containing sgRNA using Lipofectamine LTX with PLUS transfection reagent (Invitrogen, 15338100), according to the manufacturer’s instructions. After 24 h, the medium was replaced by full growth medium (DMEM). The viral preparations were collected at 48 h and 72 h post-transfection and then aliquoted and stored at −80 °C. For infection in Cas9 stable HeLa cell lines (ATG16L1 WT, KO and ATG9 WT, KO), viral titres were added to the cells in the presence of 6 µg ml^−1^ Polybrene (Sigma-Aldrich) and incubated overnight. On the following day, the medium was replaced by full growth medium and cells were incubated further before screening started.

### Western blot analysis

Cells were cultured in 6- or 12-well plates depending on experimental requirements. Cells were washed in ice-cold PBS twice, then lysed in 1× Laemmli buffer (62.5 mM Tris, pH 6.8, 2% SDS, 10% glycerol, 50 mM dithiothreitol (DTT) and 0.01% bromophenol blue) and boiled at 100 °C for 10 min or lysed in RIPA buffer (150 nM NaCl, 1% NP-40, 0.5% NaDoc, 0.1% SDS and 50 mM Tris-HCl, pH 7.4; Sigma-Aldrich) supplemented with protease and phosphatase inhibitor cocktail (Roche Diagnostics). Cells were incubated on ice for 10 min and then centrifuged at 16,000*g* for 10 min at 4 °C. Protein concentrations of supernatants were quantified using a Bradford assay kit (Bio-Rad). Then, lysates were denatured with 2× Laemmli buffer and boiled at 100 °C for 10 min. For western blot analysis, samples were subjected to SDS–PAGE separation and transferred onto PVDF membranes (Millipore, IPFL00005 or IPVH00005). PVDF membranes were blocked with 4% skim-milk in PBS containing 0.1% Tween for 1 h and incubated with primary antibody at 4 °C overnight. After washing the membrane with 0.1% Tween-PBS, the secondary antibodies were used at a dilution of 1:4,000 and incubated for 1 h at room temperature. Proteins on the membrane were visualized with direct infra-red fluorescence detection on a LI-COR Odyssey scanner or with an ECL enhanced chemiluminescence detection kit (GE Healthcare). The protein levels on the immunoblots were quantified using ImageJ program or IMAGE STUDIO Lite software.

### Immunoprecipitation

For immunoprecipitation of GFP-α-Syn A53T proteins using GFP-Trap, according to the manufacturer’s instructions (gtma-100, ChromoTek), cells were lysed with lysis buffer (10 mM Tris-HCl, pH 7.5, 150 mM NaCl, 0.5 mM EDTA and 0.5% NP-40) containing protease and phosphatase inhibitors from Roche Diagnostics for 30 min at 4 °C. The lysed cells were centrifuged at 13,000*g* for 10 min and the supernatant was transferred to a new E-tube and mixed with dilution buffer (10 mM Tris-HCl, pH 7.5, 150 mM NaCl and 0.5 mM EDTA). For input loading control, 50 µl of supernatant from each sample was mixed with 2× Laemmli buffer and then boiled at 100 °C for 10 min. The samples were further incubated with 25 µl pre-washed GFP-Trap beads for 1 h at 4 °C on a rotating surface. GFP-beads were washed five times with dilution buffer, resuspended with 2× Laemmli buffer and boiled at 100 °C for 10 min.

### Immunofluorescence

For immunofluorescence, cells on coverslips were fixed with 4% paraformaldehyde (PFA; Sigma-Aldrich) for 5 min. After washing, cells were permeabilized with 0.1% Triton X-100 for 10 min or ice-cold methanol for 15 min (for anti-PSMB4, anti-PSMA5 or anti-GFP) and blocked with 3% BSA (Thermo Scientific, BP1605-100) for 1 h. Cells were incubated with primary antibodies in blocking buffer overnight at 4 °C. After rinsing three times with PBS, cells were incubated with secondary antibodies tagged with Alexa Fluor (Molecular Probes) for 90 min at room temperature. After incubation, cells on coverslips were washed with PBS and then mounted in ProLong Gold Antifade reagent with DAPI (Invitrogen, P36931).

For aggresome detection, aggregates in cells were detected using PROTEOSTAT Aggresome detection kit (Enzo Life Sciences, ENZ-51035) following the manufacturer’s protocol. In brief, cells were fixed in 4% PFA for 30 min at room temperature and then incubated with permeabilizing solution (0.5% Triton X-100, 3 mM EDTA) on ice for 30 min with gently shaking. Cells were stained with PROTEOSTAT Aggresome Detection Reagent for 30 min at room temperature, protected from light, followed by 15 min wash in PBS. Coverslips were mounted with ProLong Gold Antifade Reagent.

Imaging was performed with LSM710 or LSM880 Zeiss confocal (×40, ×63 NA 1.4 Plan Apochromat oil-immersion lens, Carl Zeiss).

### Imaging for monitoring nucleocytoplasmic shuttling

Nucleocytoplasmic shuttling using the NLS–tdTomato–NES construct was assessed as described previously^31^. Cells were transfected with the NLS–tdTomato–NES construct (provided by J. Rothstein; Addgene cat. no. 112579). For fixed cell imaging, transfected cells were plated on glass coverslips. After drug treatments as per experimental requirements, cells were fixed with 4% PFA for 3 min. Cells on coverslips were washed with PBS and mounted in ProLong Gold Antifade reagent. For live-cell imaging, transfected cells were plated onto MatTek Petri dishes (MatTek). After adding Hoechst 33342 dye (1:5,000, Cell Signaling Technology, cat. no. 4082S) for 10 min, they were imaged at 37 °C, utilizing an LSM780 Zeiss confocal equipped with a ×63 oil-immersion lens.

### Imaging for FRAP analysis

FRAP assay was used to measure the real-time kinetics of nuclear fluorescence recovery after photobleaching in cells expressing either GFP-empty or GFP-A53T α-Syn in WT or ATG16L1-null cells. FRAP assay and analysis was performed with the approach described previously^21^.

For FRAP assay imaging, transfected cells were plated onto MatTek Petri dishes (MatTek) and then they were imaged at 37 °C, utilizing an LSM780 Zeiss confocal equipped with a ×63 oil-immersion lens. Each cell was scanned at low laser power to minimize fluorescence loss during bleaching and the region of interest (ROI) within the nucleus was then photobleached at 70% laser power with 488 nm wavelength for approximately 3 s.

For FRAP data analysis, we used two methods. The first simply assessed fluorescence recovery in the previously photobleached nuclear region. The second assessed the fluorescence of a nuclear ROI that had been previously photobleached compared with a cytoplasmic ROI (a control region) in the same cell that had not been previously photobleached; here, the fluorescence data before and after bleaching was expressed as a ratio of nuclear/cytoplasmic fluorescence.

For each analytical approach, the pre-bleach ratios were set to 100% to normalize between samples. Finally, the first post-bleach image was set to time 0, with successive time points converging toward 100% fluorescence recovery. We used this approach to normalize across different experiments, as differences in initial post-bleach mean values may be exaggerated due to the large difference in early transport rates in different experiments and a slight delay between fluorescence bleach and image capture. The recovery curves are shown each based on an average of 12–15 cells and representative of at least three independent experiments. Details are previously described^47^. To compare initial nuclear transport rates, linear regression analysis (curve-fitting) of the values from the first 32 s and the average recovery curves were performed in Excel and GraphPad Prism10.

### Synthetic lethality CRISPR/Cas9 Screen with gRNAs

To clone the gRNAs into the pKLV-PB-U6 gRNA(BbsI)-PGKpuro2ABFP (Lenti-PB) vector, pre-designed single-strand oligonucleotides with compatible overhangs for BbsI (sequences kindly provided by E. Metzakopian, UK DRI) were purchased from Merck, annealed and cloned into the BbsI site of Lenti-PB using standard restriction enzyme cloning.

Cas9 stable cells were infected with Lenti-PB vector carrying the gRNAs and BFP with about 50% infection efficiency in 96-well plates. After lentiviral gRNA infection for 3 days (starting point), the number of infected cells was monitored by BFP fluorescence using FACS (LSRFortessa instrument, BD) in the indicated intervals between starting (day 3) and finishing time point (day 12)^48^. Cells were first gated on forward (FSC-A) and side scatter (SSC-A) and then for singlets (FSC-A/FSC-H), before gating the BFP^+^ cells.

Cell viability was assessed as the percentage of BFP^+^ cells relative to the starting point (day 3 after infection). Non-targeting gRNA-transfected cells were used as a negative control. FACS-based cell number analysis was performed using FlowJo v.10.7.1 software for macOS (BD Life Sciences).

### Lactate dehydrogenase cytotoxicity assay

Lactate dehydrogenase (LDH) activity was determined with CyQUANT LDH Cytotoxicity Assay kit (Invitrogen, C20301), following the manufacturer’s instructions. To prepare controls, cells were incubated with 10 µl sterile water for the spontaneous LDH release sample or with 10 µl of 10× Lysis buffer for the maximum LDH release sample in cell culture medium for 45 min in an incubator. Then, 50 µl of each sample medium was collected and transferred to a new 96-well plate and then mixed with 50 µl reaction mixture. The plates were incubated at room temperature with light protection for 30 min. Then, 50 µl stop solution was added to each sample well to stop the reaction. LDH activity was measured by absorbance using a TECAN Spark microplate reader at 490 nm and 680 nm (as a reference wavelength).

### Incucyte live-cell imaging

For live-cell imaging of cytotoxicity, cells were incubated with CellTox Green Dye (Promega, G8741, 1:1,000 dilution) to measure cytotoxicity caused by changes of membrane integrity, according to the manufacturer’s protocol. The cell plate was placed in the Incucyte machine (Essen Bioscience, Incucyte S3 with ×10 magnification) with cell culture conditions. Images were acquired with green fluorescence (Ex, 450–490 nm; Em, 500–530 nm) and phase. The level of cell death was calculated based on dead cells with green fluorescent area (green) divided by total cells area (phase) using Incucyte, indicating that these results reflect overall cells measured with total cell area (phase). All five fields in a well were calculated per condition for each experiment.

### Detection of newly synthesized proteins

Newly synthetized proteins were detected using BONCAT (bioorthogonal non-canonical amino acid tagging) or SUnSET (surface sensing of translation) methods as previously described^26^.

For BONCAT^27^, cells were cultured in DMEM with no methionine and no cysteine (Gibco, 21013024) for 1 h and then treated with the methionine analogue AHA (Thermo Scientific, C10102) in DMEM without methionine for 4 h. To measure the levels of newly synthesized proteins in subcellular fractions by immunoblotting, nuclear/cytoplasmic fractionations were performed as described in section below with mild detergent^49^. In nuclear/cytosolic fractionations, AHA-labelled proteins were generated using the Click-iT Protein Reaction Buffer kit (Thermo Scientific, C10276) according to the manufacturer’s protocol. AHA-labelled proteins clicked with biotin alkyne (Thermo Scientific, B10185) were examined by western blotting, followed by incubation with streptavidin–Alexa Fluor 488 (Thermo Scientific, S11223). To visualize newly synthesized proteins by IF, AHA-labelled cells on glass coverslips were fixed with 4% PFA and permeabilized with 0.25% TrionX100 for 15 min. After washing with 2% BSA in PBS, AHA-labelled proteins were detected using the Click-iT Cell Reaction Buffer kit (Thermo Scientific, C10269) according to the manufacturer’s protocol. AHA-labelled proteins were clicked with either Alexa Fluor 488 Alkyne (Thermo Scientific, A10267) or Alexa Fluor 594 Alkyne (Thermo Scientific, A10275), followed by mounting with ProLong Gold Antifade with DAPI (Thermo Scientific, P36935).

For analysis of puromycin-induced foci using SUnSET methods^25^, cells were incubated with O-propargyl-puromycin (Jena Bioscience, NU-931-05) for 2 h before fixation. For analysis of ubiquitin-positive puromycin-induced foci formation, cells were incubated with 5 μg ml^−1^ puromycin for 3–4 h in full growth medium, followed by fixation in 4% PFA and staining with FK2 antibody (Millipore, 04-263). Imaging for puromycin-labelled proteins was performed as previously described^50^. In brief, O-propargyl-puromycin-labelled (Jena Bioscience, NU-931-05) cells were fixed in ice-cold methanol for 2 min at −20 °C, washed with PBS and permeabilized with 0.2% Triton X-100. Cells were stained by incubating for 30 min in 100 mM Tris, pH 8.5, 0.5 mM CuSO_4_, 20 μM Alexa Fluor 594-azide (Thermo Scientific, A10270) and 50 mM ascorbic acid, followed by washing. Coverslips were mounted with ProLong Gold Antifade Reagent.

### Nuclear and cytoplasmic fractionation

For general cell fractionation in steady-state, nuclear and cytoplasmic fractions were isolated using the NE-PER Nuclear and Cytoplasmic Extraction kit (Thermo Scientific, 78833), as described previously^13^.

To detect newly synthesized proteins in cellular fractions, subcellular fractionation was performed as described previously^49^. After incubation with AHA, cells were washed with ice-cold PBS and collected after centrifugation at 500*g* for 10 min at 4 °C. Pellets were suspended with 200 µl ice-cold lysis buffer A (NaCl 150 mM, HEPES (pH 7.4) 50 mM, digitonin (Sigma-Aldrich, D141) 25 mg ml^−1^ and hexylene glycol (Sigma-Aldrich, 112100, 1 M) containing a protease inhibitor cocktail (Roche Diagnostics, 11873580001). Samples were incubated on a rotator for 10 min at 4 °C and then centrifuged at 2,000*g* for 10 min at 4 °C. The supernatant was considered as the cytosolic fraction. Then, 200 µl ice-cold lysis buffer B (NaCl 150 mM, HEPES (pH 7.4) 50 mM, IGEPAL (Sigma-Aldrich, I7771) 1% and hexylene glycol 1 M) containing a protease inhibitor cocktail was added to the pellets and mixed by vortexing. The tubes were placed on ice for 30 min and then centrifuged at 7,000*g* for 10 min at 4 °C. The supernatant contains membrane bound organelles. The remaining pellets were resuspended with 200 µl ice-cold lysis buffer C (NaCl 150 mM, HEPES (pH 7.4) 50 mM, sodium deoxycholate 0.5%, sodium dodecyl sulfate 0.1% and hexylene glycol 1 M) containing Benzonase (Sigma-Aldrich, E1014) and protease inhibitors. For collecting nuclear fractions, samples were lysed on rotator for 30 min at 4 °C to enable complete solubilization. After centrifugation at 7,800*g* for 10 min 4 °C, supernatants containing nuclear extracts were transferred into pre-chilled new tubes. Histone H2B or Lamin B1 was used as a nuclear control and GAPDH was used as a cytosolic control.

### FACS analysis of Ub-G76V-GFP

HeLa cells stably expressing Ub-G76V-GFP, a ubiquitin fusion degradation reporter degraded by the proteasome, were previously described^26^. HeLa/Ub-G76V-GFP were treated with proteasome inhibitors for 6 h or 24 h at different concentrations. Cells were then trypsinized and GFP fluorescence was analysed using an Attune NxT Flow Cytometer (Thermo Scientific) using the BL1 (488 530/30) detector. Cells were first gated on forward (FSC-A) and side scatter (SSC-A) for P1 and then for singlets (FSC-A/FSC-H) for P2. Then, 20,000 single cells were recorded for each replicate. GFP^+^ gates were set using normal HeLa cells. The data were analysed using FlowJo software v.10.7.1.

### Bioinformatic analysis

The genetic interactions of the core autophagy genes of *Saccharomyces* *cerevisiae* (*ATG1*, *ATG2*, *ATG3*, *ATG4*, *ATG5*, *VPS30*, *ATG7*, *ATG8*, *ATG9*, *ATG10*, *ATG12*, *ATG13*, *ATG14*, *ATG16*, *ATG17*, *ATG18*, *ATG29* and *ATG31*) were downloaded from the *Saccharomyces* Genome Database (www.yeastgenome.org) using YeastMine^51^ and filtered for SL and negative genetic interactions using R software (https://www.Rproject.org/). Genes showing genetic interactions with at least five core autophagy genes were selected for further analysis. To obtain the human orthologues, the yeast genes selected were either uploaded to HumanMine^52,53^ or compared with the orthologue list downloaded from the HUGO Gene Nomenclature Committee (HGNC Database, https://www.genenames.org/)^54^.

### Image analysis

Fluorescence intensity of confocal images was measured by ImageJ. A minimum of 30 cells were examined for each condition. All experiments were repeated in at least three biological experiments (*n* = 3). To confirm previously published results, experiments were performed once with three different siRNA oligonucleotides and two different proteasome subunits or different time points and different concentrations of drugs with different inhibitors for the same mechanisms (about 30 cells in each condition). The background was fixed for all within-experiment analyses.

### Statistics and reproducibility

Significance levels for comparisons between groups were determined with an unpaired or paired two-tailed Student’s *t*-test or a one-way ANOVA, or a two-way ANOVA followed by an appropriate post hoc test for multiple comparisons using GraphPad Prism 7 and 10 (GraphPad Software) or Excel (Microsoft Office). For the two-way ANOVA, we sought to avoid situations where all values for a control condition were 1, which would result in no variance, violating assumptions for ANOVAs. Thus, we took the normalized fold changes from each experiment individually (where control data in ATG16^+^ and ATG16^−^ were originally both set to 1) and divided all data points from the normalized data by the number of conditions. The resulting numbers were used as the control values for each experiment, which were then multiplied by the fold change in each perturbation condition to derive the perturbation values. These data were used for the ANOVAs (see source data). For the graphs, we illustrate effects by setting control data in ATG16^+^ and ATG16^−^ cells to 1, so that different responses to perturbations between the different cell lines can be readily appreciated. For the one-way ANOVA, we restricted the analysis to each condition separately.

Western blots protein levels were normalized to total forms or a housekeeping protein, such as GAPDH. All data were expressed as mean ± s.e.m., as stated in figure legends and source data. *P* values <0.05 were considered statistically significant.

Sample sizes were chosen on the basis of extensive experience with the assays we have performed. No randomization was performed for cell culture experiments, as it was not necessary. But all perturbations were performed in parallel. No data were excluded from the analyses. Staining and analysis were performed in a blinded fashion. Investigators were not blinded to allocation during experiments and outcome assessment. Data distribution for parametric test was assumed to be normal but this was not formally tested.

### Reporting summary

Further information on research design is available in the Nature Portfolio Reporting Summary linked to this article.

## Online content

Any methods, additional references, Nature Portfolio reporting summaries, source data, extended data, supplementary information, acknowledgements, peer review information; details of author contributions and competing interests; and statements of data and code availability are available at 10.1038/s41556-024-01488-7.

## Supplementary information

Supplementary Information Sequential gating strategy for analysis of gRNA plasmid infected cells.

Reporting Summary

Supplementary Tables 1–6 Supplementary Table 1. Negative genetic and SL interactions with core autophagy genes. Table 2. Raw screen data in ATG16 WT and KO cells. Table 3. Raw screen data in ATG9 WT and KO cells. Table 4. Proteasome subunits or nucleoporins in negative genetic and synthetic lethal interactions with core autophagy genes. Table 5. siRNA oligonucleotides and pre-designed pLKO.1 shRNA vectors. Table 6. sgRNA oligonucleotides.

## Source data

Source Data Fig. 1 Statistical source data.

Source Data Fig. 1 Unprocessed western blots and/or gels.

Source Data Fig. 2 Statistical source data.

Source Data Fig. 2 Unprocessed western blots and/or gels.

Source Data Fig. 3 Statistical source data.

Source Data Fig. 3 Unprocessed western blots and/or gels.

Source Data Fig. 4 Statistical source data.

Source Data Fig. 4 Unprocessed western blots and/or gels.

Source Data Fig. 5 Statistical source data.

Source Data Extended Data Fig. 1 Statistical source data.

Source Data Extended Data Fig. 2 Statistical source data.

Source Data Extended Data Fig. 3 Statistical source data.

Source Data Extended Data Fig. 3 Unprocessed western blots and/or gels.

Source Data Extended Data Fig. 4 Statistical source data.

Source Data Extended Data Fig. 4 Unprocessed western blots and/or gels.

Source Data Extended Data Fig. 5 Statistical source data.

Source Data Extended Data Fig. 6 Statistical source data.

Source Data Extended Data Fig. 6 Unprocessed western blots and/or gels.

Source Data Extended Data Fig. 7 Statistical source data.

Source Data Extended Data Fig. 7 Unprocessed western blots and/or gels.

Source Data Extended Data Fig. 8 Statistical source data.

Source Data Extended Data Fig. 9 Statistical source data.

Source Data Extended Data Fig. 9 Unprocessed western blots and/or gels.

Source Data Extended Data Fig. 10 Statistical source data.

## Data Availability

All data supporting the findings of this study are available from the corresponding author upon reasonable request. Source data are provided with this paper.

## Code availability

The custom R script used in the analysis of yeast genetic interactions with core autophagy genes is available at https://github.com/adc67/YeastGeneticAnalysis.
