# Supplementary material for: Nuclear proteasomes buffer cytoplasmic proteins during autophagy compromise
Source: Nat Cell Biol. 2024 Aug 29;26(10):1691–9. doi: 10.1038/s41556-024-01488-7 (PMC11469956; doi:10.1038/s41556-024-01488-7)
Supplement: Supplementary file 1 — Sequential gating strategy for analysis of gRNA plasmid infected cells. [file 41556_2024_1488_MOESM1_ESM.pdf]

nature cell biology

Supplementary information

https://doi.org/10.1038/s41556-024-01488-7

Nuclear proteasomes buffer cytoplasmic proteins during autophagy compromise

In the format provided by the authors and unedited

Supplementary information.

Sequential gating strategy for analysis of gRNA plasmid infected cells.

a. HeLa (no Cas9)

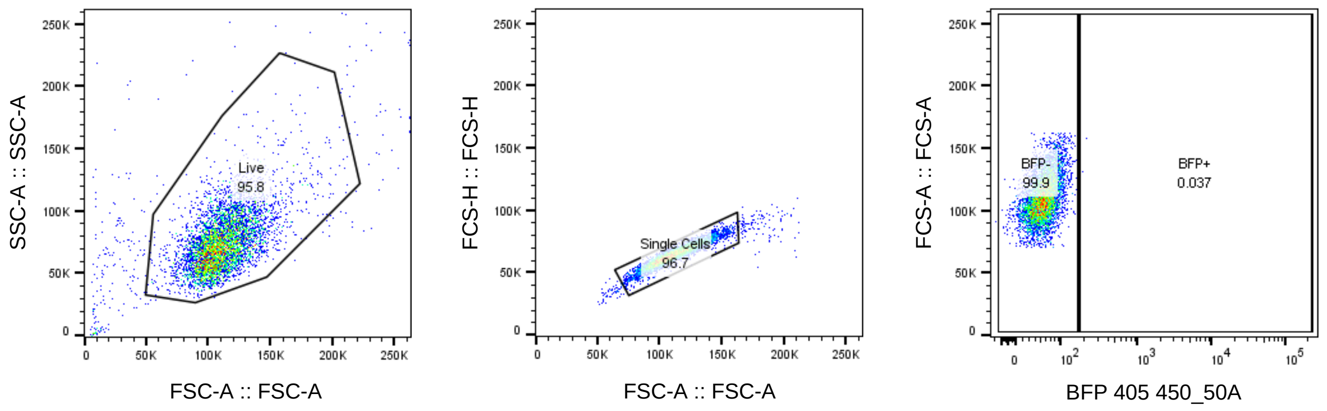

b. HeLa/Cas9 cell line with gRNA plasmid infection

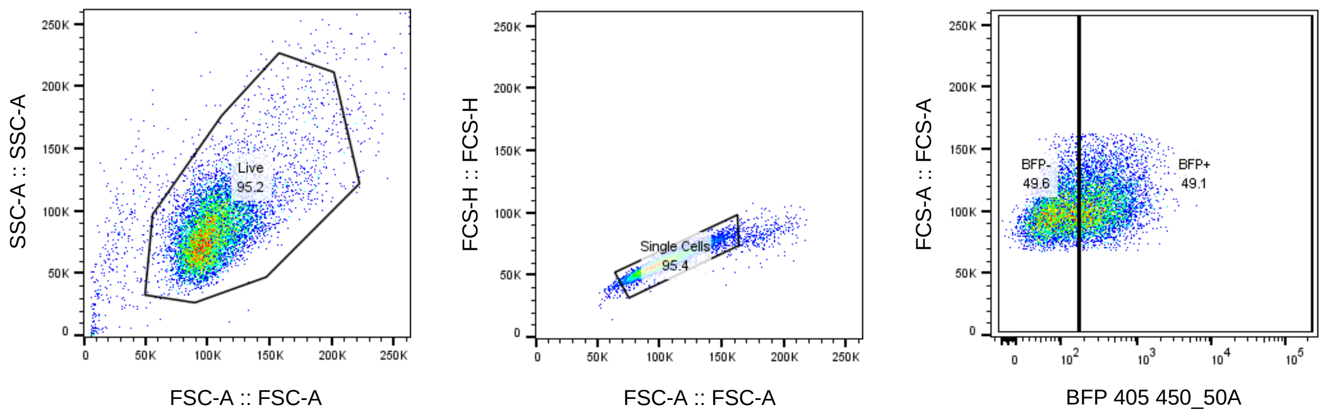

Cells were first gated on forward (FSC-A) and side scatter (SSC-A) and then for singlets (FSC-A/FSC-H), before gating the BFP+ cells. Gates for BFP positive cells population (BFP+) were defined using wild-type HeLa cells (no Cas9 cell line).
