# Supplementary material for: Nuclear proteasomes buffer cytoplasmic proteins during autophagy compromise
Source: Nat Cell Biol. 2024 Aug 29;26(10):1691–9. doi: 10.1038/s41556-024-01488-7 (PMC11469956; doi:10.1038/s41556-024-01488-7)
Supplement: Supplementary file 2 — Reporting Summary [file 41556_2024_1488_MOESM2_ESM.pdf]

nature portfolio

Corresponding author(s): David C Rubinsztein

Last updated by author(s): Jul 12, 2024

Reporting Summary

Nature Portfolio wishes to improve the reproducibility of the work that we publish. This form provides structure for consistency and transparency in reporting. For further information on Nature Portfolio policies, see our Editorial Policies and the Editorial Policy Checklist.

Statistics

For all statistical analyses, confirm that the following items are present in the figure legend, table legend, main text, or Methods section.

n/a	Confirmed	
☐	☒	The exact sample size (*n*) for each experimental group/condition, given as a discrete number and unit of measurement
☐	☒	A statement on whether measurements were taken from distinct samples or whether the same sample was measured repeatedly
☐	☒	The statistical test(s) used AND whether they are one- or two-sided<br>*Only common tests should be described solely by name; describe more complex techniques in the Methods section.*
☒	☐	A description of all covariates tested
☒	☐	A description of any assumptions or corrections, such as tests of normality and adjustment for multiple comparisons
☐	☒	A full description of the statistical parameters including central tendency (e.g. means) or other basic estimates (e.g. regression coefficient) AND variation (e.g. standard deviation) or associated estimates of uncertainty (e.g. confidence intervals)
☐	☒	For null hypothesis testing, the test statistic (e.g. *F*, *t*, *r*) with confidence intervals, effect sizes, degrees of freedom and *P* value noted<br>*Give P values as exact values whenever suitable.*
☒	☐	For Bayesian analysis, information on the choice of priors and Markov chain Monte Carlo settings
☒	☐	For hierarchical and complex designs, identification of the appropriate level for tests and full reporting of outcomes
☒	☐	Estimates of effect sizes (e.g. Cohen's *d*, Pearson's *r*), indicating how they were calculated

Our web collection on statistics for biologists contains articles on many of the points above.

Software and code

Policy information about availability of computer code

Data collection	Gels were imaged using LICOR-Odyssey apparatus using IMAGE STUDIO Lite Licor ver 5.2, Inc.<br>Confocal Carl Zeiss LSM710, LSM780 and LSM880 operated with ZEN black imaging software 2.3.<br>Plate reader Tecan SPARKControl V3.0.<br>Flow Cytometer LSRFortessa instrument (BD Life Sciences).<br>Attune NxT Flow Cytometer (ThermoFisher Scientific).<br>IncuCyte™ S3 incubated live imaging system (Essen Bioscience).<br>Microscope imaging system (Nikon Eclipse E600).<br>Data collected from the Saccharomyces Genome Database (www.yeastgenome.org) using YeastMine and filtered for synthetic lethality and negative genetic interactions using R software (R Core Team (2022), https://www.R-project.org/).<br>For human orthologues, the yeast genes selected were either uploaded to HumanMine or compared with the orthologues list downloaded from the HUGO Gene Nomenclature Committee (HGNC Database, https://www.genenames.org/).

Data analysis	IMAGE STUDIO Lite LI-COR ver 5.2, Inc and ChemiDoc Imaging Systems (1004313 CHEMIDOC-IT SYSTEM, Bio-Rad Laboratories) for gel analysis.<br>ZEN imaging software (ZEN Black 2.3 Carl Zeiss Microscopy) for microscopic image analysis.<br>Image J ver 1.54f (National Institute of Health, USA).<br>Microsoft Excel (Excel 2016 Microsoft office) and GraphPad Prism v7, v10 (GraphPad Software) for statistical analysis.<br>FlowJo Software for FACS analysis (FlowJo version 10.7.1 Software)<br>IncuCyte 2020 Software.<br>R software (R Core Team (2022), https://www.R-project.org/).<br>The custom R script used in the analysis of yeast genetic interactions with core autophagy genes is available at https://github.com/adc67/YeastGeneticAnalysis.

For manuscripts utilizing custom algorithms or software that are central to the research but not yet described in published literature, software must be made available to editors and reviewers. We strongly encourage code deposition in a community repository (e.g. GitHub). See the Nature Portfolio guidelines for submitting code & software for further information.

Data

Policy information about availability of data

All manuscripts must include a data availability statement. This statement should provide the following information, where applicable:

- Accession codes, unique identifiers, or web links for publicly available datasets
- A description of any restrictions on data availability
- For clinical datasets or third party data, please ensure that the statement adheres to our policy

All data supporting the findings of this study are available from the corresponding author on reasonable request. Source data are provided with this paper.
The data that the custom R script used in the analysis of yeast genetic interactions with core autophagy genes is available in a publicly accessible repository at https://github.com/adc67/YeastGeneticAnalysis.

Research involving human participants, their data, or biological material

Policy information about studies with human participants or human data. See also policy information about sex, gender (identity/presentation), and sexual orientation and race, ethnicity and racism.

Reporting on sex and gender	not applicable
Reporting on race, ethnicity, or other socially relevant groupings	not applicable
Population characteristics	not applicable
Recruitment	not applicable
Ethics oversight	not applicable

Note that full information on the approval of the study protocol must also be provided in the manuscript.

Field-specific reporting

Please select the one below that is the best fit for your research. If you are not sure, read the appropriate sections before making your selection.

☒ Life sciences ☐ Behavioural & social sciences ☐ Ecological, evolutionary & environmental sciences

For a reference copy of the document with all sections, see nature.com/documents/nr-reporting-summary-flat.pdf

Life sciences study design

All studies must disclose on these points even when the disclosure is negative.

Sample size	Sample sizes were chosen on the basis of extensive experience with the assays we have performed. (e.g. Son et al. Nature cell biology 2024; Son et al. Cell Metabolism 2019; Son et al. Nature communications 2020; Pavel et al. Nature communications 2021; Pavel et al. Nature communications 2018; Park et al. Cell Death and Differentiation 2022)
Data exclusions	No data were excluded from the analysis.
Replication	All experiments were repeated by at least three times and all experiments were reproducible. We used the average of each triplicate as a biological replicate for statistical analyses. To confirm the previously published results, the experiment was performed once with three different siRNA oligos and two different proteasome subunits or different time points and different concentrations of drugs with different inhibitors for the same mechanisms.
Randomization	No randomization was performed for cell culture experiments, as it was not necessary. But all perturbations were performed in parallel.

Blinding	Immunofluorescence samples were not blinded when we used imageJ software to quantify effects. Western blot analysis was not blinded as it was not possible as the gel loading order needs to be defined. SL FACS-based screen data was blinded. Proteasome activity assay was not blinded because the readout was determined by the FACS machine.

Reporting for specific materials, systems and methods

We require information from authors about some types of materials, experimental systems and methods used in many studies. Here, indicate whether each material, system or method listed is relevant to your study. If you are not sure if a list item applies to your research, read the appropriate section before selecting a response.

Materials & experimental systems

n/a	Involved in the study
☐	☒ Antibodies
☐	☒ Eukaryotic cell lines
☒	☐ Palaeontology and archaeology
☒	☐ Animals and other organisms
☒	☐ Clinical data
☒	☐ Dual use research of concern
☒	☐ Plants

Methods

n/a	Involved in the study
☒	☐ ChIP-seq
☐	☒ Flow cytometry
☒	☐ MRI-based neuroimaging

Antibodies

Antibodies used	Primary antibodies: rabbit monoclonal anti-LC3B [EPR18709] (ab192890, RRID:AB_2827794, WB 1:1000), mouse monoclonal anti-GAPDH [6C5](ab8245, RRID:AB_2107448, WB 1:5000), rabbit polyclonal anti-GFP (ab6556, RRID:AB_305564, WB 1:1000), rabbit polyclonal anti-Lamin B1 (ab16048, RRID:AB_443298, WB 1:1000), rabbit monoclonal anti-Nup98 [EPR6678] (ab124980, RRID:AB_10973030, WB 1:1000, IF 1:500), Rabbit monoclonal anti-Nup133 [EPR10808(B)] (ab155990, WB 1:1000, IF 1:500), rabbit monclonal anti-PSMD7 [EPR13517](ab181072, WB 1:1000), mouse monoclonal anti-Nuclear Pore Complex Proteins antibody [Mab414] (NPC; ab24609, RRID:AB_448181, IF 1:200), mouse monoclonal anti-KPNB1 [3E9] (ab2811, RRID:AB_2133989, WB 1:1000); rabbit polyclonal anti-KPNA2 (ab70160, RRID:AB_2133673, WB 1:1000) from Abcam; Rabbit polyclonal anti-RAN (10469-1-AP, RRID:AB_2176484, IF 1:200), rabbit polyclonal anti-Lamin B1 (12987-1-AP, RRID:AB_2136290, WB 1:1000), rabbit polyclonal anti-H2B (15857-1-AP, RRID:AB_10664929, WB 1:1000), mouse monoclonal anti-GFP [1E10H7] (66002-1-Ig, RRID:AB_11182611, WB 1:2000, IF 1:1000), rabbit polyclonal anti-GFP (50430-2-AP, RRID:AB_11042881, WB 1:2000), rabbit polyclonal anti-LC3 (14600-1-AP, RRID:AB_2137737, WB 1:3000) from Proteintech; Rabbit anti-K48-linkage polyubiquitin (8081, RRID:AB_10859893, WB 1:1000), rabbit polyclonal anti-PSMA5 (2457,RRID:AB_823611, IF 1:500), rabbit monoclonal anti-ATG16L1 [D6D5] (8089, RRID:AB_10950320, WB 1:1000) from Cell Signaling; rabbit polyclonal anti-ATG16L1 (MBL, PM040, RRID:AB_1278757, WB 1:1000); Mouse anti-puromycin (MABE343, RRID:AB_2566826, 1:1000), mouse-anti-Polyglutamine-Expansion (MAB1574, RRID:AB_94263, IF 1:200), anti-Ubiquitinylated proteins (FK2) (04-263, RRID:AB_612093, IF 1:1000) from Millipore; Rabbit polyclonal anti-AKIRIN2 (Atlas Antibodies, HPA064239, RRID:AB_2685222, WB 1:1000, IF 1:500); Mouse monoclonal anti-C6ORF166 [3D9] (AKIRIN2) (Abnova, H00055122-M01, RRID:AB_894020, IF 1:500); Mouse monoclonal anti-PSMB4 [H-3] (Santacruz, sc-390878, IF 1:100); Mouse monoclonal anti-HA.11 clone 16B12 (Covance, MMS-101P, RRID:AB_10064068, IF 1:500).<br>Secondary Antibodies: anti-mouse (#NA931V, RRID:AB_772210) and anti-rabbit (#NA934V) horseradish peroxidise (HRP)-conjugated secondary antibodies (GE Healthcare).<br>For immunoflourescence, goat-anti-mouse Alexa Fluor 488 (#A11029, RRID:AB_2534088, 1:400), 555 (#A21147, RRID:AB_1500897, 1:400) and 594 (#A11032, RRID:AB_2534091, 1:400), goat-anti-rabbit Alexa Fluor 488 (#A32731, RRID:AB_2633280, 1:400), 555 (#A21428, RRID:AB_141784, 1:400), streptavidin-Alexa Fluor 488 (#S11223, 1:2000), Alexa Fluor 488-Alkyne (A10267) and Alexa Fluor 594-Alkyne (A10275) from ThermoFisher.
Validation	All antibodies used in this study were purchased from commercial vendors who had validated specificity in human cells/ mouse tissues for the specific assays (Western blot, immunoprecipitation and/or immunofluorescence), described on data sheets and online. We have confirmed that the LC3 antibody does not give an LC3-II band in autophagy null cells.

Eukaryotic cell lines

Policy information about cell lines and Sex and Gender in Research

Cell line source(s)	HeLa (human cervical epithelium) (ATCC; #CCL-2; CVCL_0030), HEK293FT (human embryonic kidney cell line; Invitrogen, #R70007), striatal cell lines derived from wild-type HTT Q7/Q7 (Coriell Institute #CH00097), heterozygous HTT Q7/Q111 (Coriell Institute #CH00096), homozygous HTT Q111/Q111 knock-in mice (Coriell Institute #CH00095).<br>Primary fibroblasts from 3 unaffected controls (Ctrl, Coriell Institute, #GM04711 (Cont1); #GM04729 (Cont2), #GM04865 (Cont3)), 5 Huntington's disease patients (HD, Coriell Institute #GM21757 (HD1), #GM0485 (HD3), GM04287 (HD4), #GM21756 (HD5); polyQ17/80 #HD30501 (HD2) was a kind gift from Ferdinando Squitieri, Huntington, and Rare Diseases Unit, Fondazione IRCCS Casa Sollievo della Sofferenza Research Hospital, Italy).<br>Autophagy-deficient ATG16L1 CRISPR knockout (ATG16L1 KO) HeLa cells and control cells (ATG16L1 WT) and ATG9 CRISPR knockout (ATG9 KO) HeLa cells and control HeLa celss (ATG9 WT) (Runwal, G., 2019).<br>The G3 line of induced pluripotent stem cells (iPSCs) previously derived from WTC11 (Cont iPSCs) were kindly provided by Michael E. Ward (National Institute of Neurological Disorders and Stroke, National Institutes of Health, Bethesda, Maryland) (Fernandopulle, M.,2018). For HTT125Q iPSCs, iPSCs were generated from peripheral blood mononuclear cells donated by a

	juvenile HD patient originally carrying 125CAGs (HTT125Q). HTT125Q iPSCs were a kind gift from Gabriel Balmus (UK DRI) (Goold, R., 2021).
Authentication	All the cell lines were ordered from ATCC, ECACC or Coriell Institute with authentication.<br>The cell lines were ordered from ATCC, Horizon or Coriell Institute with authentication.<br>HeLa authenticatd by ATCC (by Short Tandem Repeat (STR) profiling; FTA barcode:STRA1466)<br>HEK293FT were purchased from Invitrogen.<br>ATG16L1 CRISPR knockout (ATG16L1 KO) HeLa cells and control cells (ATG16L1 WT) were authenticated by Western blot analysis with ATG16L1 antibody.<br>ATG9 CRISPR knockout (ATG9 KO) HeLa cells and control cells (ATG9 WT) were authenticated by Western blot analysis with ATG9 antibody shown in Runwal, G., 2019 PMID: 31300716.<br>Cas9 cell lines were authenticated by assessing their FACS profile.<br>HTT Q7/Q7, Q7/111 and Q111/Q111 striatal cell lines were authenticated by Western blot analysis with Huntingtin antibody (different migration of Huntingtin protein caused by the presence of polyQ expansion) and polyQ antibody verified in Wrobel L, 2022 PMID: 35842429; Ashkenazi A, 2017 PMID: 28445460.<br>Huntington's disease patient fibroblasts were authenticated by Western blot analysis with Huntingtin antibody (different migration of Huntingtin protein caused by the presence of polyQ expansion) and polyQ antibody verified in Wrobel L, 2022 PMID: 35842429; Ashkenazi A, 2017 PMID: 28445460.<br>HTT125Q iPSCs were authenticated by immunostaining with polyQ antibody and verified in Goold R, 2021, PMID: 34469738.<br>Ub-G76V-GFP HeLa cells were authenticated by assessing FACS profile verified in Wrobel L, 2022 PMID: 35842429.
Mycoplasma contamination	All the cells were regularly tested using EZ-PCR Mycoplasma Test Kit (Biological Industries; cat#20-700-20). Cells used in this study were mycoplasma negative.
Commonly misidentified lines<br>(See ICLAC register)	no commonly misidentified cell lines were used in the study.

Flow Cytometry

Plots

Confirm that:

☒ The axis labels state the marker and fluorochrome used (e.g. CD4-FITC).

☒ The axis scales are clearly visible. Include numbers along axes only for bottom left plot of group (a 'group' is an analysis of identical markers).

☒ All plots are contour plots with outliers or pseudocolor plots.

☒ A numerical value for number of cells or percentage (with statistics) is provided.

Methodology

Sample preparation	For Cas9 stable cell line, ATG16L1 WT and KO and ATG9 WT and KO were transduced virus carrying LentiCas9 blast (pKLV-Cas9 blast). Cas9 stable cell line (the pool) was tested for Cas9 cutting efficiency with a lentiviral vector encoding BFP, GFP and a sgRNA against GFP. The percentage of BFP+/GFP- (edited) to BFP+/GFP+ (total transduced) cells was analysed in Cas9 negative and Cas9 positive cells by FACS using the LSRFortessa flow cytometer (BD).<br>Cas9 stable cells were infected with Lenti-PB vector carrying the gRNAs and BFP with about 50% infection efficiency in 96-well plates. After lentiviral gRNA infection for 3 days (starting point), the number of infected cells was monitored by BFP fluorescence using FACS (LSRFortessa instrument, BD) in the indicated intervals between starting (Day3) and finishing time point (Day12).(Pavlou, S., 2023, Tzelepis, K., 2016)<br>For Ub-G76V-GFP HeLa cells, cells were treated with various compounds and then trypsinised and fluorescence was analysed using Attune NxT Flow Cytometer (ThermoFisher Scientific) using the BL1 (488 530/30) detector (for GFP tagged Ub-G76V).
Instrument	Flow Cytometer (LSRFortessa instrument, BD), Attune NxT Flow Cytometer (ThermoFisher Scientific)
Software	FlowJo version 10.7.1 Software
Cell population abundance	Cells are stably expressing Cas9 (LentiCas9 blast (pKLV-Cas9 blast)) infected with Lenti-PB vector carrying the gRNAs and BFP with about 50% infection efficiency (Pavlou S. 2023 PMID: 36894612, Tzelepis K. 2016 PMID: 27760321).<br>For Ub-G76V-GFP HeLa cells, cells are stably expressing indicated proteins with EGFP tag (Wrobel L, 2022 PMID: 35842429).
Gating strategy	Cells were first gated on forward (FSC-A) and side scatter (SSC-A) and then for singlets (FSC-A/FSC-H), before gating the BFP+ cells. Cell viability was assessed the percentage of BFP+ cells relative to the starting point (Day3 after infection). Non-targeting gRNA transfected cells were used for negative control. Gates were set using normal HeLa cells. (Pavlou S. 2023 PMID: 36894612, Tzelepis K. 2016 PMID: 27760321)<br>For Ub-G76V-GFP HeLa cells, cells were first gated on forward (FSC-A) and side scatter (SSC-A) for P1 and then for singlets (FSC-A/FSC-H) for P2. 20,000 single cells were recorded for each replicate. GFP+ gates were set using normal HeLa cells, shown in Wrobel L, 2022 PMID: 35842429.

☒ Tick this box to confirm that a figure exemplifying the gating strategy is provided in the Supplementary Information.
