# Supplementary material for: Nuclear proteasomes buffer cytoplasmic proteins during autophagy compromise
Source: Nat Cell Biol. 2024 Aug 29;26(10):1691–9. doi: 10.1038/s41556-024-01488-7 (PMC11469956; doi:10.1038/s41556-024-01488-7)

Unprocessed blots Extended Data Fig. 3. Square indicates the section shown in the Extended Data Figures. Molecular weight is shown on the left.

Extended Data Fig 3k

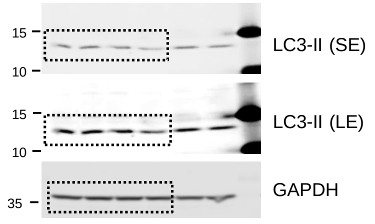

Supplement: Supplementary file 16 — Unprocessed western blots and/or gels. [file 41556_2024_1488_MOESM16_ESM.pdf]
