# Supplementary material for: Nuclear proteasomes buffer cytoplasmic proteins during autophagy compromise
Source: Nat Cell Biol. 2024 Aug 29;26(10):1691–9. doi: 10.1038/s41556-024-01488-7 (PMC11469956; doi:10.1038/s41556-024-01488-7)

Unprocessed blots Extended Data Fig. 6. Square indicates the section shown in the Extended Data Figures. Molecular weight is shown on the left.

Extended Data Fig 6d

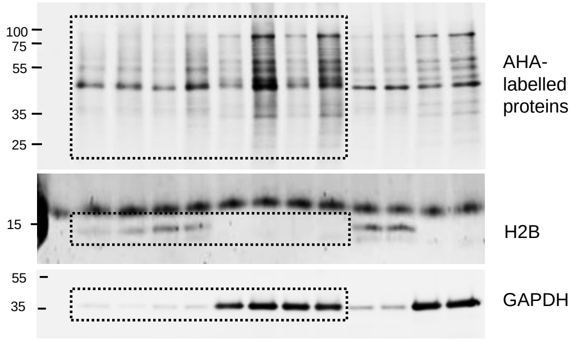

Extended Data Fig 6g

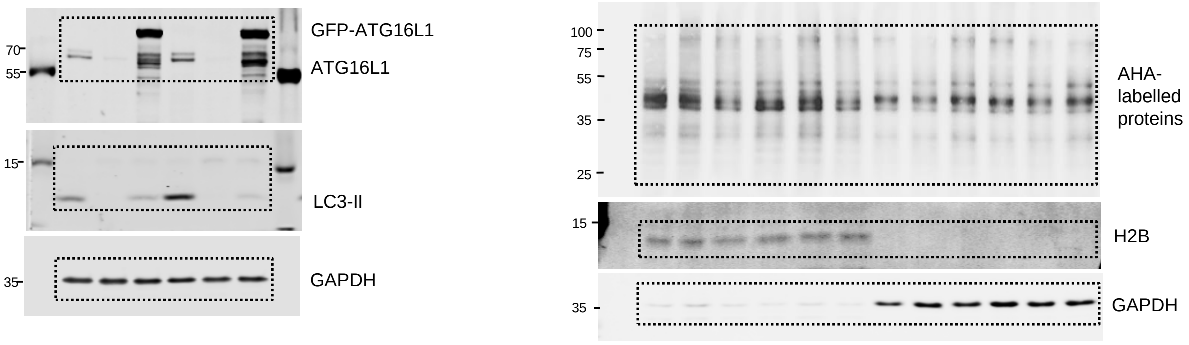

Supplement: Supplementary file 21 — Unprocessed western blots and/or gels. [file 41556_2024_1488_MOESM21_ESM.pdf]
